# Magnetoelectric BaTiO_3_/CoFe_2_O_4_ Thin‐Film Meshes for Neuronal Differentiation

**DOI:** 10.1002/advs.77180

**Published:** 2026-08-15

**Authors:** Mathieu Mirjolet, Hao Ye, Abderrahim Lahlahi Attalhaoui, Donghoon Kim, Vitaly Pustovalov, Elric Zhang, Marc Tudela‐Pi, Bastian Gebauer, Juan Bautista Carda Castelló, Anton Guimerà‐Brunet, Josep Puigmartí‐Luis, Xiang‐Zhong Chen, Minsoo Kim, Salvador Pané

**Affiliations:** ^1^ Multi‐Scale Robotics Lab Institute of Robotics and Intelligent Systems ETH Zurich Zurich Switzerland; ^2^ Department of Inorganic and Organic Chemistry Universitat Jaume I Castellón de la Plana Spain; ^3^ Instituto de Microelectronica de Barcelona (IMB‐CNM, CSIC) Bellaterra Spain; ^4^ Centro de Investigación Biomédica en Red en Bioingeniería, Biomateriales y Nanomedicina (CIBER‐BBN) Madrid Spain; ^5^ Departament de Ciència de Materials i Química Física Institut De Química Teòrica i Computacional Universitat De Barcelona Barcelona Spain; ^6^ Institució Catalana de Recerca i Estudis Avançats (ICREA) Barcelona Spain; ^7^ International Institute for Intelligent Nanorobots and Nanosystems College of Intelligent Robotics and Advanced Manufacturing State Key Laboratory of Photovoltaic Science and Technology Shanghai Frontiers Science Research Base of Intelligent Optoelectronics and Perception, Institute of Optoelectronics Fudan University Shanghai People's Republic of China; ^8^ Zhejiang Key Laboratory of Extreme Environment Functional Materials Yiwu Research Institute of Fudan University Yiwu; ^9^ Department of Robotics and Mechatronics Engineering DGIST Daegu Republic of Korea

**Keywords:** cell differentiation, freestanding thin films, magnetoelectric, multiferroic, neural progenitor cells, pulsed laser deposition

## Abstract

Magnetoelectric composites offer a powerful route for remotely converting magnetic stimuli into local electrical cues, with promising biomedical applications in electrical stimulation. In strain‐mediated magnetoelectric heterostructures, however, efficient coupling depends strongly on crystallinity, interface quality, and mechanical boundary conditions. Epitaxial thin films provide excellent crystallinity and well‐defined interfaces, but substrate clamping suppresses strain transfer and limits magnetoelectric performance. Here, we report a freestanding magnetoelectric mesh based on an epitaxial BaTiO_3_/CoFe_2_O_4_ bilayer and demonstrate its use for magnetic‐field‐driven neural progenitor cell differentiation. By systematically relaxing the mechanical boundary condition from substrate‐clamped films to polydimethylsiloxane (PDMS)‐transferred structures and ultimately to freestanding meshes, we show a progressive enhancement in the magnetoelectric coupling coefficient. This enhancement is directly reflected in improved neural progenitor differentiation, establishing magnetoelectric transduction as a key driver of the observed cellular response. Our results show that engineering mechanical compliance in epitaxial magnetoelectric platforms can substantially improve biofunctional stimulation, providing a design strategy for next‐generation cell culture, differentiation, and wireless stimulation systems.

## Graphical abstract

We engineer an epitaxial BaTiO_3_/CoFe_2_O_4_ magnetoelectric mesh that overcomes boundary‐induced mechanical clamping. Progressively reducing the mechanical constraint, from substrate‐clamped to PDMS‐transferred and ultimately freestanding, enhances magnetic‐to‐electric transduction and strengthens magnetoelectric coupling. This tunable coupling, in turn, influences neural progenitor cell differentiation, providing a strategy for optimizing magnetoelectric biointerfaces.

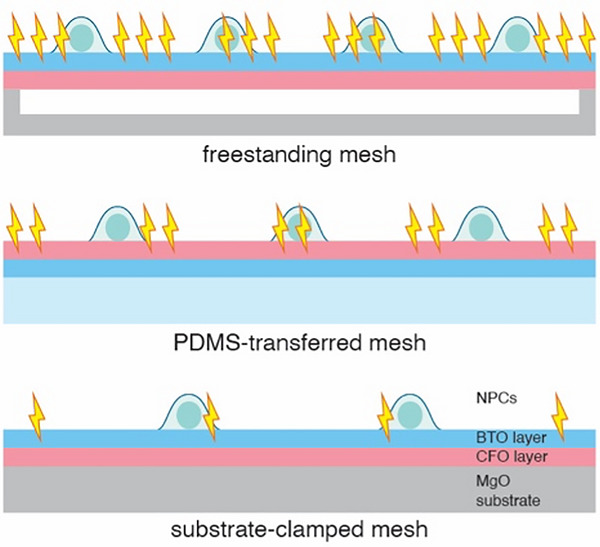

## 1 Introduction

Functional oxide ceramics are generally associated with brittleness and limited deformability. Yet, when reduced to nanoscale dimensions and integrated into engineered bilayer architectures, they can display mechanical responses that differ markedly from those of their bulk counterparts. Recent studies on epitaxial BaTiO_3_/CoFe_2_O_4_ (BTO/CFO) bilayers have revealed an unusual combination of superelastic behavior, transferability to compliant supports, and retention of functional integrity [1, 2, 3]. These observations suggest that nanoscale ceramic bilayers may provide a promising platform for biointerfaces capable of operating under mechanically dynamic conditions, including those imposed by adherent cells. In such systems, mechanical adaptability is not merely a structural advantage, but a potentially important factor for maintaining functional performance in biological environments.

In this context, BTO/CFO bilayers are particularly attractive, as the combination of a piezoelectric BTO layer and a magnetostrictive CFO layer gives rise to magnetoelectric (ME) coupling capable of converting external magnetic fields into localized electrical signals through strain‐mediated transduction [4, 5, 6, 7]. This wireless transduction capability makes ME composites broadly attractive for biomedical technologies where remote actuation without wired connections or implanted power sources is desirable. ME composites have therefore attracted growing interest for applications including cell differentiation [8, 9, 10, 11], neurostimulation [12, 13, 14, 15, 16, 17, 18, 19], drug delivery [20, 21, 22, 23, 24, 25], tissue regeneration [26, 27, 28], and physiological signal acquisition [29]. For these applications, however, the effectiveness of the material platform depends not only on the magnitude of the ME response, but also on its ability to interface mechanically with soft and dynamically active biological systems.

Different composite architectures have been explored to meet these requirements. Core–shell magnetoelectric nanoparticles (MENPs), especially CFO‐BTO systems, have been widely investigated for biomedical use because of their minimal invasiveness and their ability to be magnetically guided to target sites [11, 12, 13, 15, 18, 19, 22, 23, 30, 31, 32]. They have shown promise in neural stimulation, cancer treatment, and spinal cord repair [12, 23, 27]. However, their practical implementation remains limited by imperfect core–shell interfaces, random particle orientation, reduced macroscopic coupling, and difficulties in localization or retrieval after treatment. Laminated ME composites can offer improved control over electric‐field orientation and may benefit from resonance‐enhanced responses [14, 28, 29, 33, 34], but they generally rely on mechanically bonded interfaces, which restrict strain transfer and can increase invasiveness and device complexity.

Among ME composite platforms, thin‐film architectures offer a particularly attractive balance between reduced dimensions and strong interfacial coupling. In epitaxial magnetostrictive/piezoelectric bilayers, coherent interfaces can promote efficient strain transfer, making them promising for high‐performance ME transduction [35, 36, 37, 38, 39, 40, 41]. At the same time, the functional response of such systems is strongly influenced by mechanical boundary conditions. In conventional substrate‐clamped films, deformation of the magnetostrictive layer is constrained by the rigid support, suppressing strain transfer to the piezoelectric layer and thereby limiting the overall ME response [42]. Recent work on released and transferred epitaxial BTO/CFO heterostructures has pointed to the importance of mechanical constraint in governing coupling behavior, suggesting that the controlled release of substrate clamping could provide an effective route for enhancing ME functionality [1, 2, 3].

Motivated by this opportunity, here we introduce epitaxial ME mesh structures that combine strong interfacial coupling with mechanical flexibility and tunable mechanical constraint. The mesh architecture was selected to provide high surface coverage while preserving structural robustness and enabling different degrees of mechanical release. By optimizing mesh geometry and processing conditions, we realized homogeneous BTO/CFO ME meshes in three boundary‐dependent configurations: substrate‐clamped, polydimethylsiloxane (PDMS)‐transferred, and fully freestanding. These structures preserve epitaxial quality while progressively relaxing substrate clamping, resulting in tunable strain transfer and ME coupling behavior. To evaluate this effect across length scales, the magnetoelectric coupling coefficient (MECC) was quantified locally by piezoresponse force microscopy and macroscopically through ME‐induced electrolyte conductance measurements. The ME response was found to depend strongly on the mechanical boundary conditions, with the fully freestanding meshes exhibiting the highest coupling, followed by the PDMS‐transferred and substrate‐clamped configurations. Finally, neural progenitor cells (NPCs) cultured on these ME meshes exhibited differentiation under alternating magnetic stimulation, and the biological response followed the same trend as the MECC. These results identify mechanical boundary conditions as a key design parameter linking ME coupling in epitaxial ceramic composites to bioelectrical stimulation and cell differentiation.

## 2 Results

**FIGURE 1:**
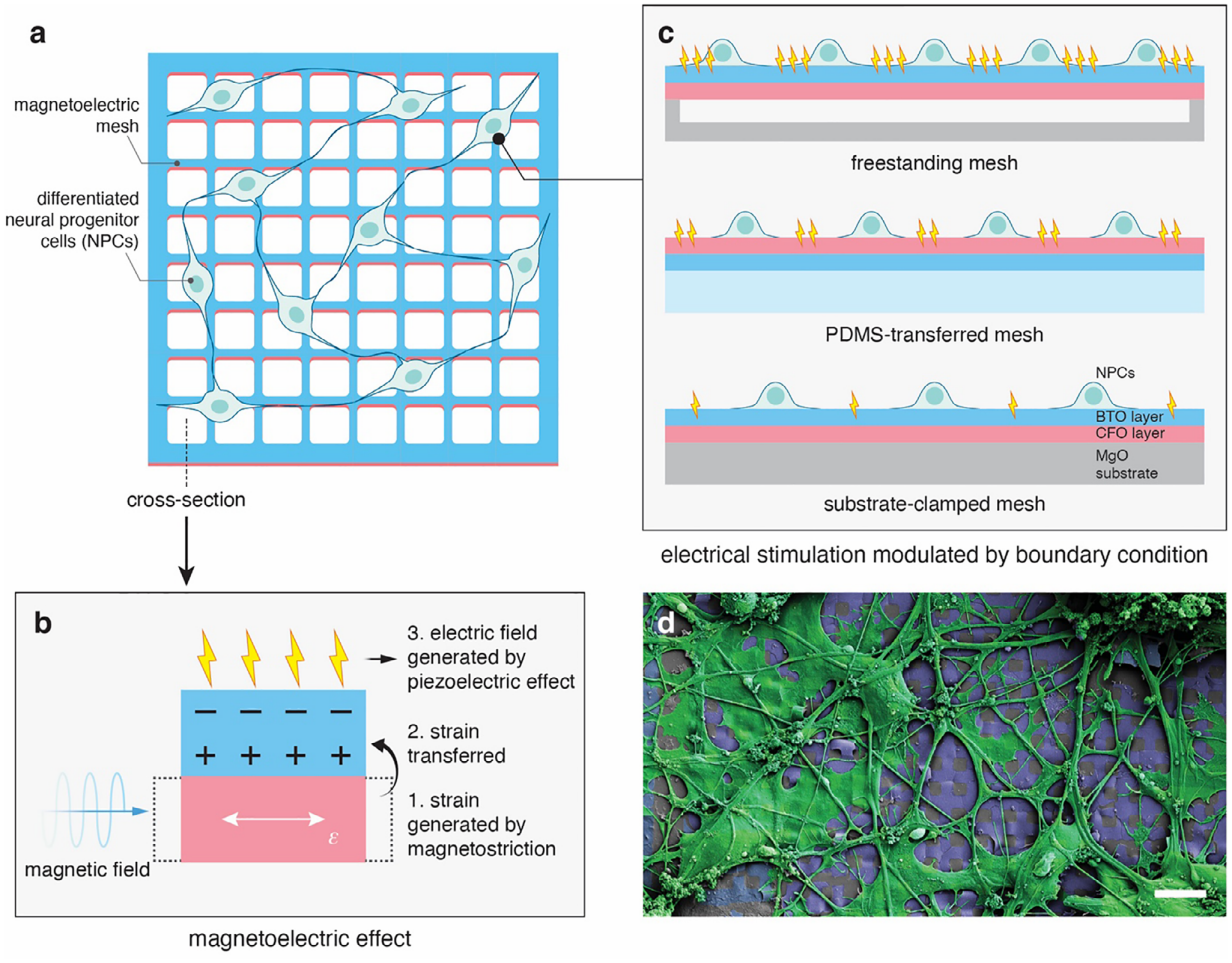
Magnetoelectric (ME) mesh for cell differentiation. (a) Schematic illustration of the configuration of the ME mesh and neural progenitor cells (NPCs). (b) Mechanism of the strain‐mediated ME effect. (c) Boundary condition‐dependent ME effect and corresponding cell differentiation performance. (d) Scanning electron microscopy (SEM) image of differentiated cells cultured on the PDMS‐transferred ME mesh (scale bar: 10 µm).

Figure 1 illustrates the overall concept of ME thin‐film meshes that induce magnetoelectric stimulation to promote NPC differentiation. NPCs are cultured on the ME meshes (Figure 1a), where the application of an external magnetic field induces electric field modulation that stimulates the cells. The applied magnetic field deforms the magnetostrictive layer, and the resulting strain is transferred to the piezoelectric layer, leading to the generation of an electric field (Figure 1b).

This strain‐mediated ME effect is strongly influenced by the substrate, as substrate‐induced clamping compromises strain generation in the magnetostrictive layer. Building on our previous findings, we designed three ME mesh configurations with distinct mechanical boundary conditions: freestanding, PDMS‐transferred, and substrate‐clamped (Figure 1c). Among them, the freestanding mesh produced the most effective magnetoelectric stimulation, resulting in enhanced neural progenitor cell (NPC) differentiation. A representative result of ME‐induced NPC differentiation is shown in Figure 1d.

### 2.1 Design and Fabrication of ME Thin‐Film Mesh

**FIGURE 2:**
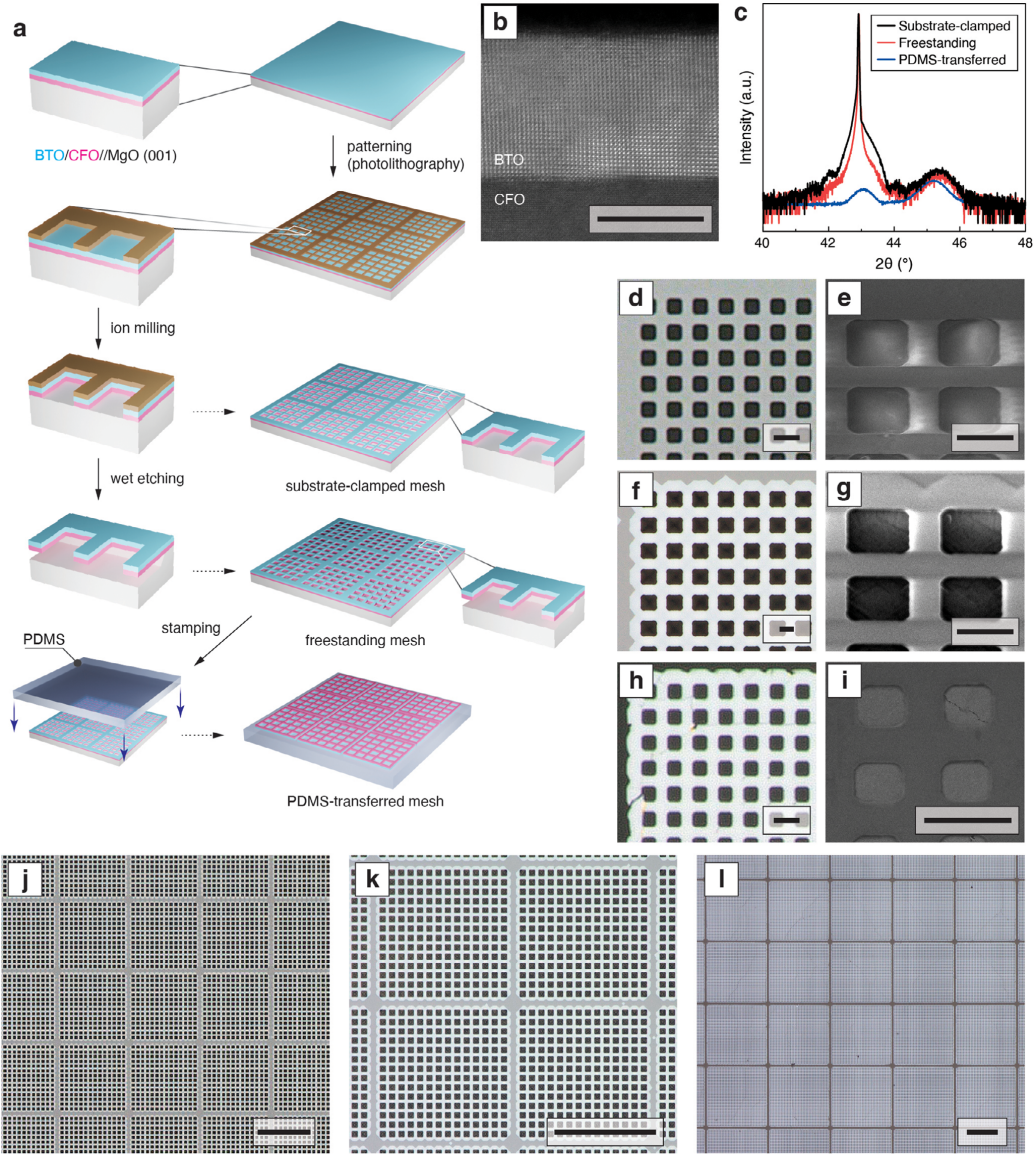
Fabrication procedure of ME meshes. (a) Schematic illustration of the fabrication process. In each stage (after ion milling, wet etching, and stamping), the substrate‐clamped mesh, freestanding mesh, and PDMS‐transferred mesh are obtained, respectively. (b) Transmission electron microscopy (TEM) cross‐sectional image of the BTO/CFO bilayer of substrate‐clamped ME mesh (scale bar: 5 nm). (c) X‐ray diffraction (XRD) θ‐2θ spectra around the 002 MgO reflection for the substrate‐clamped, freestanding, and PDMS‐transferred meshes. (d) Optical image and (e) tilted SEM image of the substrate‐clamped mesh. (f) Optical image and (g) tilted SEM image of the freestanding mesh. (h) Optical image and (i) tilted SEM image of the PDMS‐transferred mesh. (j, k) Optical images of the freestanding mesh and (l) PDMS‐transferred mesh showing their scalable fabrication. Scale bars in (d–i) represent 5 µm, and those in (j–l) represent 100 µm.

Figure 2a shows the overall fabrication process for the substrate‐clamped, freestanding, and PDMS‐transferred meshes. First, BTO/CFO (15 nm/15 nm) bilayers were deposited onto 10 × 10 mm^2^ MgO substrates using pulsed laser deposition (PLD). The PLD process enabled epitaxial bilayers with a coherently strained interface (Figure 2b,c). Subsequently, photolithography and etching were employed to define the mesh patterns. A positive photoresist was applied and patterned using a photolithographic mask corresponding to the mesh design. After exposure and development, the unprotected regions of the film were physically etched by Ar ion milling. The remaining photoresist was subsequently removed, completing the preparation of substrate‐clamped mesh (Figure 2d,e). The substrate‐clamped mesh was then immersed in a sodium bicarbonate‐saturated solution, which selectively etched the MgO substrate to release the freestanding mesh (Figure 2f,g). Finally, the freestanding mesh was transferred onto a PDMS host substrate via a stamping process, resulting in a PDMS‐transferred mesh (Figure 2h,i). The scalability of this fabrication process was demonstrated by the successful preparation of large‐area meshes, as shown in the optical images of the freestanding (Figure 2j,k) and PDMS‐transferred (Figure 2l) configurations.

A regular mesh pattern was selected as the structural design for this study. Since wet etching of the MgO substrate is relatively slow, a pattern that allows the etchant to access the substrate surface efficiently was preferred to complete the process within a reasonable timescale. To achieve this, the mesh was designed with thin stripes oriented along the [100] and [010] crystallographic directions, enabling uniform and efficient substrate removal during etching. In addition, the regular mesh pattern provides a mechanically robust geometry that minimizes the influence of residual stress compared with more complex designs. Here, the mesh was further divided into several submeshes to prevent a single fracture from propagating and damaging the entire structure. As a result, this design and fabrication strategy allows the production of large‐scale freestanding meshes with minimal fracture, representing a clear advantage over continuous membrane structures.

Mesh design parameters include stripe width, spacing, and submesh size. They are critical for increasing fabrication yield, mesh structure reliability, and NPC stimulation efficiency. For example, a smaller stripe width allows faster substrate etching and increases structural flexibility (Figure S1); however, it reduces the amount of active material per unit area, potentially limiting the efficiency of NPC stimulation. In the case of spacing, it influences both the mechanical stability of the mesh and overall stimulation efficiency. Last, a smaller submesh size minimizes the propagation of fractures but also decreases the available stimulation area due to the gap required for dividing the mesh into submeshes.

Considering these factors, the mesh geometry was empirically optimized to achieve minimum fracture of meshes upon release from the MgO substrate while retaining as much ME material as possible. We found that larger stripes with smaller spacing increased the ME material coverage but were limited by manufacturability and structural stability issues, including significantly longer MgO etching times. Conversely, smaller stripes with wider spacing yielded a higher proportion of intact meshes but substantially reduced the amount of active ME material. Details of the optimization process are provided in Note S1 and Figures S2–S6. The optimized mesh parameters were determined to be a stripe width of 2–3 µm and a spacing of 5 to 9 µm, ensuring that approximately 60% of the ME material remained on the substrate. The entire sample surface was divided into 60 × 60 submeshes, each approximately 150 × 150 µm^2^, separated by 10 µm gaps that left the ME thin film clamped to the substrate. This design helps relieve stress accumulation and minimize the number of fractured meshes during release. Finally, using the optimal photolithographic mesh design (Figure S7), the clamped, freestanding, and PDMS‐transferred meshes were successfully implemented (Figure S8).

To support the experimental optimization of mesh geometry, computational simulations were performed using COMSOL Multiphysics based on a bilayer BTO/CFO mesh design. The design parameters, including stripe width (1, 2, and 3 µm), spacing (4, 8, and 12 µm), and number of patterns (2 × 2, 4 × 4, and 6 × 6), were systematically varied to evaluate structural deformation and stress distribution. The primary factors of interest were the maximum deflection and the stress concentration at the corners. Simulation results revealed that increasing the stripe width led to higher stress but lower deflection. Increasing the spacing and the number of patterns resulted in slightly higher stress concentrations and greater deflection. Therefore, narrower stripe widths, smaller spacing, and fewer patterns (i.e., smaller submesh) are preferable for maintaining structural integrity. These findings highlight a trade‐off between mechanical stability and coverage (e.g., ME material covering area per total surface area), suggesting that stripe widths of 2–3 µm and spacing of 5–9 µm are acceptable parameters for achieving both structural reliability and cell stimulation efficiency.

### 2.2 Boundary Condition‐Dependent Properties of ME Thin‐Film Mesh

#### 2.2.1 Microstructures

X‐ray diffraction *θ‐2θ* scan in clamped BTO/CFO bilayer reveals that the bilayers show high crystallinity with 001 crystalline orientation (Figure 2c and Figure S13). The 002 reflection of CFO is a small shoulder located at *2θ* ≈ 43.1°, accompanied by Laue fringes and corresponding to a c‐axis *c*_CFO_ = 4.186 Å, as expected for a CFO strained layer onto an MgO (4.021 Å) substrate. On the other hand, the 002 BTO reflection is located at *2θ* ≈ 45.4°. The corresponding c‐axis, *c*_BTO_ = 3.996 Å, denotes the prevalence of a‐ and b‐domains. Long‐range *θ‐2θ* scan (Figure S13c) also confirms the highly crystalline nature of both layers.

Atomic force microscopy images of the as‐deposited BTO/CFO bilayer (Figure S14) show a predominantly flat surface with a root‐mean‐square (RMS) roughness of 0.36 ± 0.02 nm, indicating mostly layer‐by‐layer growth. The smooth morphology suggests good epitaxial quality, although partial strain relaxation is expected in the BTO layer due to the lattice mismatch with CFO.

To ensure that the integrity and high crystallinity of the BTO/CFO mesh were preserved during the fabrication process, we performed the *θ‐2θ* scans of clamped, freestanding, and PDMS‐transferred meshes (Figure 2c). The results show that all samples maintained high crystallinity. No discernible change in lattice parameters was detected between the clamped and freestanding meshes. In contrast, the mesh transferred onto PDMS clearly shows peak shifts, with c‐axis values of *c*_CFO_ = 4.199 Å and *c*_BTO_ = 4.009 Å. The elongation of these c‐axes indicates strain relaxation following complete release from the MgO‐induced tensile strain. The freestanding mesh does not go through the same relaxation, as it is still anchored to the MgO substrate at its edges.

Surface morphology of the clamped and PDMS‐transferred meshes (Figures S15 and S16) confirms that the 2‐µm stripe width and 5‐µm spacing were well patterned. Cross‐sectional views of the transferred mesh show a BTO/CFO thickness of ≈30 nm, indicating preferential etching of the MgO substrate and preservation of film integrity throughout the process. The mesh is therefore cleanly transferred onto the PDMS substrate, demonstrating that the etching steps did not degrade the film. More details on the microstructure characterization can be found in Note S2.

The high epitaxial quality of the BTO/CFO bilayer was confirmed by high‐angle annular dark‐field scanning transmission electron microscopy (HAADF‐STEM) throughout the boundary conditions. The TEM lamella image shows a uniform thickness across the bilayer structure (Figure S17a). Magnified TEM images show that the epitaxial BTO/CFO bilayer is well preserved in both the clamped configuration (Figure 2b and Figure S17b) and the freestanding configuration (Figure S17c). TEM images of the PDMS‐transferred configuration (Figure S18) also reveal a well‐preserved bilayer with crystallinity comparable to that of the freestanding and clamped configurations. No degradation in film crystallinity or thickness is observed, confirming that the fabrication process does not adversely affect film quality.

#### 2.2.2 Magnetoelectric Properties

**FIGURE 3:**
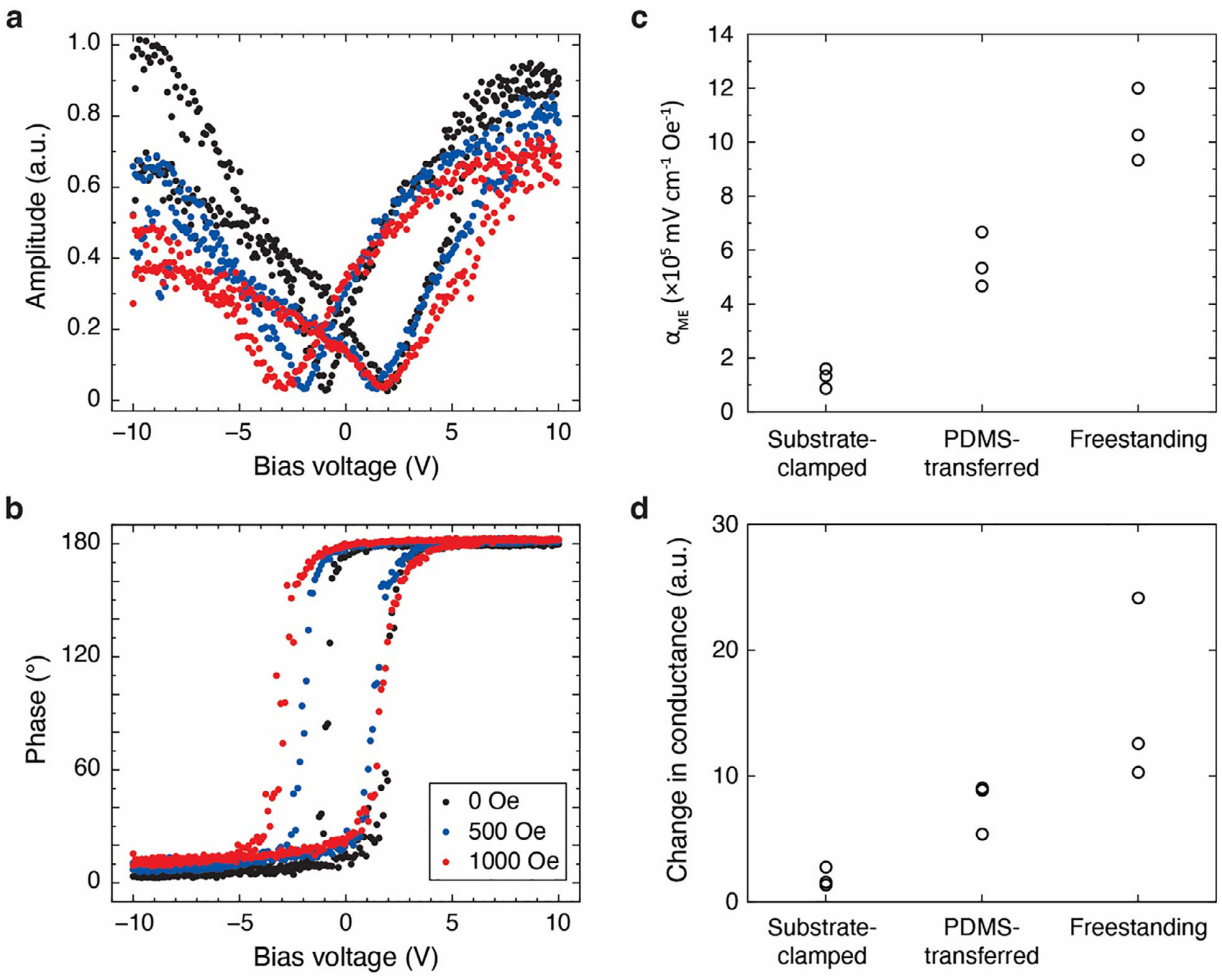
Boundary condition‐dependent ME properties. (a, b) Amplitude and phase loops of freestanding ME meshes under varying external magnetic field application, measured by piezoresponse force microscopy (PFM). (c) Comparison of the magnetoelectric coupling coefficient extracted from PFM measurements for meshes with different boundary conditions. (d) Comparison of the change in electrolyte conductance induced by the ME effect of meshes with different boundary conditions, measured using an electrochemical setup.

Using both piezoresponse force microscopy (PFM) and electrochemical measurements, we evaluated the magnetoelectric properties of meshes under different boundary conditions, namely clamped, PDMS‐transferred, and freestanding. Figure 3a,b shows the PFM amplitude and phase loops, respectively, for a freestanding mesh. Increasing the external magnetic field produces a horizontal shift in both amplitude and phase loops, enabling estimation of the MECC [43]. The MECC is calculated using Equation (1).

(1) $$\alpha_{ME}=\frac{\Delta E}{\Delta H}=\frac{\Delta V/t}{\Delta H}$$

where *ΔE* is the measured out‐of‐plane electric field that can be estimated by the shift of the center of the hysteresis loop *ΔV*, *t* is the thickness of the ferroelectric layer (here 15 nm), and *ΔH* is the applied in‐plane magnetic field. In Figure 3b, the center of the phase loop shifts from +0.554 V to −0.348 and −0.695 V as the applied magnetic field increases from 0 Oe to 500 and 1000 Oe, respectively. This shift in the center of the phase loop was consistently observed across multiple cycles of magnetic field application (Figure S19). From the shift observed between 0 and 500 Oe (Figure 3b), and using Equation (1), we obtained a MECC of α_*ME*_ ≈ 12 × 10^5^ mV cm^−1^ Oe^−1^. Overall, an average MECC of α_*ME*_ ≈ 10.5 × 10^5^ mV cm^−1^ Oe^−1^ is observed for the freestanding meshes. The average MECC decreases to α_*ME*_ ≈ 5.6 × 10^5^ mV cm^−1^ Oe^−1^ in PDMS‐transferred meshes, and to α_*ME*_ ≈ 1.3 × 10^5^ mV cm^−1^ Oe^−1^ in substrate‐clamped meshes (illustrative measurements can be found in Figure S20). Figure 3c summarizes the MECC variation across the different boundary conditions. Freestanding, PDMS‐transferred, and substrate‐clamped meshes correspond to low, intermediate, and high clamping levels, respectively. The MECC exhibits a clear trend, displaying an inverse dependence on the degree of clamping. These values and trends are in close agreement with our previous findings [2].

To correlate this observation, we similarly compared the set of thin‐film meshes in an electrochemical conductance measurement setup. The setup consists of a small cuvette filled with a thin layer of saline solution with 4 electrodes arranged in a standard 4‐point probe setup. When the ME meshes are placed in solution, an applied magnetic field produces a high‐strength, localized electric field that interferes with the free‐ion mobility in solution, changing the conductance in a measurable fashion. As the strength of the electric field corresponds with the strength of the magnetoelectric effect, it is expected that the mesh with the lowest degree of clamping would correspond with the largest change in conductance, which was observed experimentally (Figure 3d). The conductance change appears to scale proportionally with the MECC, which matches the trend observed with PFM measurements.

### 2.3 ME Mesh‐Based NPC Differentiation

#### 2.3.1 Biocompatibility

**FIGURE 4:**
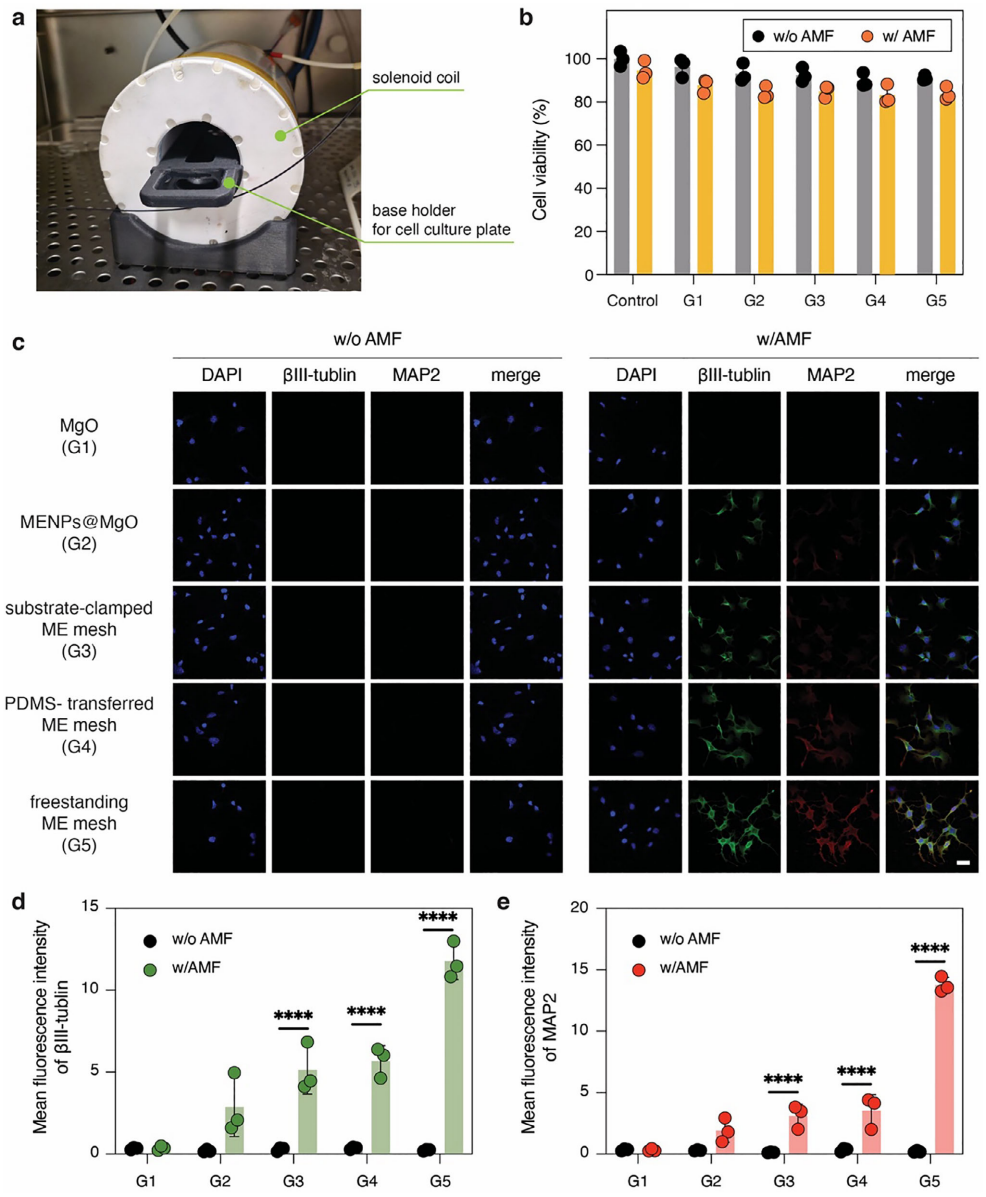
ME mesh‐based NPC differentiation. (a) Experimental setup for magnetoelectric stimulation of NPCs to evaluate biocompatibility and differentiation performance. (b) Evaluation of biocompatibility on ME meshes under varied boundary conditions, showing maintained cell viability. (c–e) Immunofluorescence analysis of ME meshes showing the expression of neuronal markers. Samples were stained for βIII‐tubulin and MAP2 to identify differentiated neurons. Quantitative assessment was performed through mean fluorescence intensity (MFI) across all samples (*n* = 3, means ± s.e.m.). ^****^ *p* < 0.0001. Scale bar: 8 µm.

We investigated whether ME meshes can drive cell differentiation, especially neural progenitor cell (NPC), and first verified their biocompatibility. We prepared six experimental groups, including (i) NPCs (control), (ii) NPCs + MgO substrate (G1), (iii) NPCs + MENPs dispersed on MgO substrate (G2), (iv) NPCs + substrate‐clamped ME mesh (G3), (v) NPCs + PDMS‐transferred ME mesh (G4), and (vi) NPCs + freestanding ME mesh (G5). Each experimental group was further divided into subgroups that were either exposed to or not exposed to an alternating magnetic field (AMF). The samples were placed at the center of a solenoid coil with an internal diameter of 5 cm, operating in a resonant tank configuration (Figure 4a). This solenoid coil generated an AMF with an amplitude of 20 mT and a frequency of 1.18 kHz. The AMF was applied for 2 h, twice daily, over two consecutive days. Figure 4b quantifies NPC viability in each group with and without AMF. Both the presence of the ME materials and AMF led to only modest decreases relative to control. Group G4 with the CFO layer facing the cells showed the lowest viability, consistent with the inherent cytotoxicity of CFO. All groups nevertheless remained above 80%, which exceeds the ISO 10993–5 2009 cytotoxicity threshold and indicates overall biocompatibility within the experimental timeframe.

#### 2.3.2 Differentiation of NPCs Using ME Meshes

To evaluate the differentiation potential of NPCs under ME stimulation, we employed the same AMF protocol as described in Section 2.3.1. The AMF exposure lasted for 2 h, twice daily, over two consecutive days to activate the ME effect and enhance NPC differentiation. For the control, the same mass of MENPs was dispersed onto the MgO substrate (G2) to compare the efficiency of ME meshes in promoting cell differentiation. Figure 4c shows immunofluorescence imaging conducted after AMF stimulation, revealing pronounced neuronal differentiation of NPCs cultured on ME meshes, evidenced by enhanced expression of βIII‐tubulin and microtubule‐associated protein 2 (MAP2). Quantitative analysis of mean fluorescence intensity (MFI) revealed significant increases in both neuronal markers compared to unstimulated controls (Figure 4d,e and Figure S22). Under the three respective boundary conditions, AMF application resulted in increases in MFI of 17.3‐, 21.0‐, and 48.0‐fold for βIII‐tubulin, and 17.4‐, 20.7‐, and 63.3‐fold for MAP2. Comparison of SEM images showing cell differentiation on the PDMS‐transferred mesh (Figure 1d and Figure S21a, with AMF; Figure S21b, without AMF) further confirms effective neural induction mediated by the ME mesh under AMF stimulation. Differences in neuronal marker expression were also observed among ME meshes with varied boundary conditions. An increasing trend was observed from substrate‐clamped to PDMS‐transferred to freestanding meshes, consistent with the trend in the ME coupling coefficient, indicating that the boundary state influences the efficiency of ME‐mediated neural induction. Notably, the ME meshes exhibited slightly enhanced differentiation outcomes compared to MENPs.

## 3 Discussion

This study demonstrates the significance of high‐quality, efficiently engineered multiferroic thin‐film meshes for biomedical applications, particularly for stimulating and promoting the differentiation of NPCs. In BTO/CFO heterostructures, the piezoelectric polarization of BTO is modulated through the magnetostrictive response of CFO under external magnetic fields, thereby tuning the out‐of‐plane polarization. This tunability alters the surface charge environment of the BTO layer and, consequently, influences local ionic behavior at the cell interface. Because NPC differentiation is closely linked to Ca^2+^ channel activity and ion migration, the ability of ME meshes to electrically modulate this ionic landscape provides a powerful pathway for controlling cellular behavior. To enable this functionality, we developed the multiferroic heterostructure, optimized the mesh geometry, and established robust fabrication processes. Boundary‐condition control clearly demonstrates the role of the ME effect in NPC differentiation, with freestanding meshes exhibiting the strongest response. Moreover, the PDMS‐transferred design offers a more stable and application‐ready device configuration.

The enhanced NPC differentiation observed here may be related to local magnetoelectric charge modulation at the cell‐material interface, which could induce membrane depolarization, activate Ca^2+^‐permeable ion channels, promote Ca^2+^ influx, and subsequently trigger electrostimulation‐associated signaling pathways involved in neuronal differentiation. This proposed mechanism is consistent with previous work using the same induced pluripotent stem cell (iPSC)‐derived NPCs and CFO‐BTO magnetoelectric system, in which AMF stimulation was shown to enhance intracellular Ca^2+^ influx, while Ca^2+^ channel blockade markedly reduced the expression of neuronal and astrocytic markers [27]. In the present study, we primarily focus on the materials design and boundary‐condition‐dependent enhancement of magnetoelectric coupling in BTO/CFO meshes. However, further studies are needed to fully elucidate the biological mechanisms underlying the response observed in this mesh system.

Optimizing the mesh design has been important for achieving magnetoelectrically induced NPC differentiation. While a stripe width of 2–3 µm has been finally selected to ensure a high packing ratio, mechanical stability could be further improved with narrower stripes in the 1–2 µm range. Specifically, a 1.5 µm stripe width reliably produced a fully preserved sample without any submesh fracture (Figure S1), indicating that thinner stripes combined with larger spacing promote high structural stability and robustness. This geometry, however, was not chosen because the remaining BTO/CFO material after patterning was relatively low (≈22%) compared with the final selected designs (≈60%). Moving forward, an application‐specific balance between mechanical reliability and ME material coverage will be important to optimize, depending on the targeted performance requirements of the mesh.

Interestingly, for the optimal geometry, the fracture rate remains below 5%, and in multiple fabrication batches we achieve a 100% yield of fully freestanding submeshes. Several factors can increase the likelihood of submesh fracture, including intrinsic film defects (e.g., point defects, microcracks), imperfections introduced during patterning (such as dust particles, photoresist edge beads, or non‐uniform coating), and variations in the release steps, particularly the substrate etching rate and total etching time. Understanding and minimizing these factors are essential for improving fabrication reliability.

The proposed fabrication method has proven to be a highly efficient and versatile approach for producing freestanding BTO/CFO structures. We demonstrate a broad range of mesh geometries with systematically varied parameters. In addition, our previous work has shown that this method can realize diverse architectures, from simple stripe patterns to complex kirigami designs, that exhibit enhanced mechanical properties (e.g., superelasticity and shape memory) and improved magnetoelectric performance due to reduced substrate clamping [1, 2, 3, 42]. This geometrical versatility allows for further variation in design, ranging from 2D to 3D, thereby enabling a wider range of functional applications (e.g., implantable cell‐stimulation devices adaptable to curved surfaces). Here, a simple 2D mesh design was selected for its high surface coverage and mechanical stability. However, the same methodology could be applied to membranes of other shapes that, for instance, could guide NPC growth in specific directions, ultimately producing a desired shape for the generated neuronal structure.

In comparing the MECC under different boundary conditions, analyses of the PFM hysteresis loops (e.g., amplitude and phase) show an increasing trend of MECC, which is enhanced from substrate‐clamped samples to PDMS‐transferred and freestanding meshes. When the magnetic field is applied, the hysteresis loop generally shifts along the voltage axis as the magnetostrictive strain affects the built‐in electric field. For example, if the magnetostrictive strain favors polarization pointing up, the phase loop will shift so that switching to the disfavored direction requires a larger opposite voltage. Here, we observed that changes in the boundary conditions altered the degree of the PFM loop shift, likely due to the clamping effect restricting magnetostriction. These variations in MECC are consistent with the results from the electrochemical method, which quantifies ionic conductance, and ultimately lead to differences in magnetoelectric cell‐differentiation performance.

Despite these achievements, several tasks remain before these multiferroic materials can be fully implemented in practical biological applications. While fully freestanding meshes exhibit strong ME coupling, they are impractical for biomedical use due to their fragility and handling challenges. Conversely, substrate‐clamped films provide mechanical stability, but display limited ME responses because of substrate‐induced constraints. The PDMS‐transferred mesh thus serves as a practical alternative, offering mechanical flexibility and biocompatibility while maintaining effective ME functionality suitable for bioelectronic applications.

Freestanding ME meshes have exhibited superior NPC differentiation performance compared to MENPs, which may be attributed to the use of commercial CFO nanoparticles and sol–gel synthesis methods. That is, interface control and geometry optimization were not considered for enhancing the ME properties. With further refinement of the synthesis process, the ME properties of MENPs are expected to improve. Moreover, ME thin films and MENPs can be regarded as complementary platforms, each offering distinct advantages and addressing different application needs. The selection between the two should be guided by the specific requirements of the target application, such as spatial precision, stimulation strength, and fabrication scalability.

While this study establishes a robust fabrication strategy and demonstrates the fundamental performance of freestanding BTO/CFO magnetoelectric meshes, several avenues remain open for future investigation. First, the influence of mesh geometry on magnetoelectric performance was not systematically explored. Whether specific shapes (e.g., geometries incorporating larger openings) could enhance magnetostriction and thereby increase the MECC, as suggested in previous work [42], remains an important question. Also, a deeper understanding of stress‐release mechanisms during etching and transfer, as well as microstructural evolution such as crack initiation and propagation, will be essential for improving mesh yield and long‐term reliability. Together, these findings establish the promise of multiferroic ME meshes as versatile, wireless bioelectronic interfaces and lay a solid foundation for their advancement toward neuromodulation and regenerative medicine applications.

## 4 Experimental Section

### 4.1 Thin Film Deposition

BTO/CFO bilayers were deposited by PLD onto 10 × 10 mm^2^ (001) MgO substrates (Crystal GmbH) using a 248 nm KrF excimer laser. A Neocera PLD system was used, which provides improved thickness uniformity over a large substrate area due to its relatively large target‐to‐substrate distance (approximately 75 mm).

Prior to deposition, substrates were ultrasonically cleaned in acetone and isopropanol, followed by a light ion‐milling step (with 250 mA of beam current and 600 V of voltage) for 1 min to remove contamination (from carbonates, hydroxides, hydrocarbons), shortly before introducing the substrate into the PLD chamber. The 15 nm CFO layer was then deposited at T_S_ = 550°C and PO_2_ = 10 mTorr, using a laser fluence of 1.8 J cm^−2^ at 5 Hz; followed by the BTO layer deposition at T_S_ = 750°C and PO_2_ = 200 mTorr, with laser fluence of 1.5 J cm^−2^ at 4 Hz.

### 4.2 Mesh Patterning

Mesh patterning was done by photolithography, using AZ1505 positive photoresist, followed by dry etching of the undesired parts by ion‐milling (Oxford IonFab 300 Plus). This dry etching was done in 15 short cycles of 20 s at 500 mA beam current (and 600 V beam voltage), with resting steps of 90 s between cycles to limit photoresist hardening. After physical etching, the resist was stripped by acetone and oxygen plasma at 600 W for 3 min. For freestanding meshes, the samples were placed in sodium bicarbonate‐saturated solution for about 48–72 h to etch the MgO substrate. After thorough rinsing with deionized (DI) water and isopropyl alcohol (IPA), the samples were dried using a critical point drier (from Tousimis). Samples were either left as such, to create freestanding meshes, or transferred to PDMS by stamping method.

The mesh structure was designed by opening square regions in the continuous bilayer, resulting in grids of 90°‐intertwined stripes of 2–3 µm width whose centers are separated by 5–9 µm. It was designed in a way that only regions ≈150 × 150 µm^2^ are freestanding, to avoid excessive material loss in case of fracture, by leaving clamped wider stripes of ≈10 µm width in between the freestanding regions. All in all, the ME bilayer covers ≈60% of the total substrate surface (10 × 10 mm^2^).

For the PDMS‐transferred meshes, the stamping was done by a dry‐transfer stamping process. A flat PDMS substrate was manually placed onto the freestanding mesh on the MgO substrate and subsequently peeled away in a single motion, transferring the mesh onto the PDMS.

### 4.3 Nanoparticle Preparation

The CFO‐BTO core–shell nanoparticles were synthesized using a sol–gel method. For the core material (CFO), commercial cobalt ferrite nanoparticles (CAS: 12052‐28‐7) were used. To deposit the BTO shell, two precursor solutions (A and B) were first prepared. Solution A was prepared by dissolving 1.736 g of barium acetate (CAS: 543‐80‐6) in 5 mL of acetic acid (CAS: 64‐19‐7), which was slightly heated to facilitate dissolution. The mixture was stirred for 1 h and then cooled to room temperature. Solution B was prepared by mixing 10 mL of 2‐methoxyethanol (CAS: 109‐86‐4) with 1 mL of acetylacetonate (CAS: 123‐54‐6) under vigorous stirring for 5 min, followed by the dropwise addition of 2.776 mL of titanium(IV) butoxide (CAS: 5593‐70‐4). Once Solution A had cooled, it was added dropwise to Solution B to form Solution C, which was stirred for an additional 30 min. A suspension of 250 mg of CFO dispersed in 1 mL of DI water was added to Solution C at a 1:10 v/v ratio and shaken for 1 h. The resulting mixture was then dried overnight on a hotplate at 100°C without stirring. The dried powder was subsequently annealed in an oven at 900°C for 2 h. All chemicals were obtained from Sigma–Aldrich and used as received.

### 4.4 Structural Analysis

The crystalline structure of the ME meshes was analyzed by X‐ray diffraction (XRD, Panalytical X'Pert MRD) employing Cu Kα_1_ radiation (λ = 1.5406 Å). Surface morphology and topographical features were examined by atomic force microscopy (AFM, Bruker Dimension FastScan) in semi‐contact mode. Optical microscopic images were captured using a Keyence VHX‐X1 digital microscope. For transmission electron microscopy (TEM) measurements, lamellae of the BTO/CFO meshes were prepared by focused ion beam (FIB) in situ lift‐out method with a Helios 5 UX system (Thermo Scientific, USA). For atomic structure analysis, the lamellae were examined using a double Cs‐corrected GrandARM microscope (JEOL, Japan), operated at 300 kV in scanning transmission electron microscopy (STEM) mode.

### 4.5 Magnetoelectric Coupling Property Evaluation: Piezoresponse Force Microscopy

Ferroelectric properties of BTO/CFO samples were investigated by piezoelectric force microscopy (PFM, NT‐MDT). Freestanding meshes were transferred onto Au‐coated Si substrates. PDMS‐transferred meshes were transferred to Au‐coated PDMS. Clamped meshes were deposited onto a MgO substrate first coated with a TiN or BLSO bottom electrode. For magnetoelectric coupling evaluation, PFM measurements were acquired at different dc magnetic fields ranging from 0 to 1000 Oe, and the MECC *α*_ME_ was defined by Equation (1). Three independent samples were measured for each boundary condition.

### 4.6 Magnetoelectric Coupling Property Evaluation: Electrochemical Setup

ME coupling evaluated via conductance measurements was performed using a custom‐assembled cuvette with four electrodes arranged in a standard four‐point probe setup, similar to previously reported experiments [44]. After placement of the sample, the cuvette was filled with phosphate‐buffered saline (PBS), diluted 1:4 (v/v) with DI water, sufficient to fully cover the sample. After sufficient time to stabilize the conductance level, a 350‐mT magnetic field was applied to the sample, causing an immediate change in conductance. This shift was then compared against the baseline value to calculate the normalized change in conductance.

### 4.7 Cell Culture

Human iPSC‐derived neural progenitor cells (NPCs, Merck, SCC035) were expanded on Matrigel‐coated culture vessels. Matrigel was thawed on ice, diluted 1 to 50 in ice‐cold Dulbecco's Modified Eagle Medium, applied to flasks, and incubated overnight at 4°C. Immediately before seeding, the coating solution was aspirated, and vessels were equilibrated with prewarmed ENStem‐A Neural Expansion Medium (Merck, SCM004a). Cryopreserved NPCs were then rapidly thawed at 37°C, transferred to a sterile 15 mL conical tube, and gently diluted with prewarmed medium to minimize osmotic stress. Cells were pelleted by centrifugation, the supernatant was removed, and the pellet was resuspended in fresh expansion medium. Viability and cell number were determined, and cells were seeded onto the prepared Matrigel‐coated vessels. Cultures were maintained at 37°C in a humidified 5% CO_2_ atmosphere with expansion medium exchange every two days. At confluence, NPCs were dissociated with Accutase (Merck, SCR005), counted, and replated into freshly coated T75 flasks for continued expansion.

### 4.8 Biocompatibility Test

The biocompatibility was evaluated through cell viability assays using MTT over a 48‐h period. Each ME mesh was sterilized by UV irradiation overnight, followed by immersion in a 10% penicillin–streptomycin and Amphotericin B solution. The samples were then thoroughly rinsed with phosphate‐buffered saline (PBS) and placed individually into wells of a culture plate (e.g., ibidi, 81201) for NPC seeding. Before cell seeding, the chamber was coated with Matrigel using the same protocol and incubated overnight. NPCs were cultured at a defined cell density (5000 cells/well) for subsequent MTT [3‐(4,5‐dimethylthiazol‐2‐yl)‐2,5‐diphenyltetrazolium bromide] analysis. Six experimental groups were prepared: (i) NPCs (control), (ii) NPCs + MgO substrate (G1), (iii) NPCs + MENPs dispersed on MgO substrate (G2), (iv) NPCs + substrate‐clamped ME mesh (G3), (v) NPCs + PDMS‐transferred ME mesh (G4), and (vi) NPCs + freestanding ME mesh (G5). Each group was further divided into subgroups with or without exposure to an alternating magnetic field (AMF). Each subgroup contained 3 biologically independent samples (*n* = 3) prepared and analyzed under the same experimental conditions. Twenty‐four hours post‐seeding, following the previously established AMF stimulation protocol [27], samples were positioned within a solenoid coil (5 cm inner diameter) operating in a resonant tank configuration. An alternating magnetic field with an amplitude of 20 mT and a frequency of 1.18 kHz was applied for 2 h twice daily over two consecutive days. After treatment, cell viability was assessed via MTT assay using a tetrazolium salt concentration of 3 mg mL^−1^. Absorbance was measured at 570 nm with a reference wavelength of 630 nm using a Varioskan Flash multimode microplate reader. Mean absorbance values from triplicate wells were used to calculate cell viability according to:

$$Cell\;viability\;of\;NPC\;\left[\%\right]=\frac{OD_{sample}-OD_{blank}}{OD_{control}-OD_{blank}}\;\times 100$$

where *OD*_sample_, *OD*_blank_, and *OD*_control_ represent the optical densities of treated NPCs, blank medium (without cells), and untreated control NPCs (without ME mesh or AMF exposure), respectively.

### 4.9 NPC Differentiation

The neurostimulation capability of the ME meshes was evaluated using the same experimental groups described in Section 4.8 by assessing their ability to promote NPC differentiation. Following the AMF stimulation protocol detailed in Section 4.8, an AMF with an amplitude of 20 mT and a frequency of 1.18 kHz was applied for 2 h, twice daily, over two consecutive days. After stimulation, cells were fixed with 4% paraformaldehyde for 10 min at room temperature. Cells were permeabilized with 0.5% Triton X‐100 for 30 min at room temperature, then blocked with 2% bovine serum albumin for 30 min to reduce nonspecific binding. For neuronal marker detection, cells were incubated overnight at 4°C with primary antibodies against βIII‐tubulin (Sigma–Aldrich T2200) and MAP2 (Proteintech 67015‐1). Secondary antibodies were fluorescein isothiocyanate (FITC)‐conjugated goat anti‐rabbit IgG (Sigma–Aldrich AP132F) and tetramethylrhodamine isothiocyanate (TRITC)‐conjugated goat anti‐mouse IgG (Sigma–Aldrich AP503R), each applied for 1 h, followed by several washes and nuclear counterstaining. The images were captured on a Zeiss LSM 880 Airyscan confocal microscope.

## Author Contributions

**Xiang‐Zhong Chen**: supervision, conceptualization, methodology, formal analysis, project administration, resources, funding acquisition, writing – original draft, writing – review and editing. **Abderrahim Lahlahi Attalhaoui**: data curation, validation, investigation, formal analysis. **Hao Ye**: methodology, validation, formal analysis, data curation, visualization, writing – review and editing. **Juan Bautista Carda Castelló**: supervision, writing – review and editing, funding acquisition, resources. **Marc Tudela‐pi**: investigation, methodology. **Anton Guimerà‐brunet**: methodology, investigation, funding acquisition, supervision, formal analysis. **Mathieu Mirjolet**: conceptualization, methodology, data curation, investigation, validation, formal analysis, supervision, writing – original draft, writing – review and editing. **Josep Puigmartí‐luis**: methodology, supervision, funding acquisition, investigation, formal analysis, project administration, resources. **Elric Zhang**: supervision, formal analysis, writing – review and editing. **Donghoon Kim**: conceptualization, methodology, supervision, writing – review and editing. **Salvador Pané**: conceptualization, methodology, supervision, funding acquisition, project administration, resources, writing – original draft, writing – review and editing. **Minsoo Kim**: conceptualization, methodology, data curation, investigation, formal analysis, supervision, writing – original draft, writing – review and editing, validation. **Bastian Gebauer**: validation, investigation, data curation, formal analysis, visualization, methodology. **Vitaly Pustovalov**: validation, data curation, investigation.

## Funding

This work was funded by the Swiss National Science Foundation (Grant No. 213113).

## Conflicts of Interest

The authors declare no conflicts of interest.

## Supporting information




**Supporting File**: advs77180‐sup‐0001‐SuppMat.pdf.

## Data Availability

The data that support the findings of this study are available from the corresponding author upon reasonable request.
