# Supplementary material for: Magnetoelectric BaTiO3/CoFe2O4 Thin‐Film Meshes for Neuronal Differentiation
Source: Adv Sci (Weinh). 2026 Aug 15:e77180. Online ahead of print. doi: 10.1002/advs.77180 (PMC13477256; doi:10.1002/advs.77180)
Supplement: Supplementary file 1 [file ADVS-9999-e77180-s001.pdf]

Supporting Information

Magnetoelectric $BaTiO_3/CoFe_2O_4$ Epitaxial Thin-Film Meshes for Neuronal Differentiation

Mathieu Mirjolet, Hao Ye, Abderrahim Lahlahi Attalhaoui, Donghoon Kim, Vitaly Pustovalov, Elric Zhang, Marc Tudela-Pi, Bastian Gebauer, Juan Bautista Carda Castelló, Anton Guimerà-Brunet, Josep Puigmartí-Luis, Xiang-Zhong Chen, Minsoo Kim*, Salvador Pané**

M. Mirjolet, H. Ye, A. Lahlahi-Attalhaoui, D. Kim, V. Pustovalov, E. Zhang, B. Gebauer, M. Kim, S. Pané
Multi-Scale Robotics Lab, Institute of Robotics and Intelligent Systems, ETH Zurich, 8092 Zurich, Switzerland
E-mail: haoyeh@ethz.ch, vidalp@ethz.ch

A. Lahlahi-Attalhaoui, J. B. Carda
Department of Inorganic and Organic Chemistry, Universitat Jaume I, 12071 Castellón de la Plana, Spain

M. Tudela-Pi, A. Guimerà-Brunet
Instituto de Microelectronica de Barcelona (IMB-CNM, CSIC), Campus UAB, 08193 Bellaterra, Spain
Centro de Investigación Biomédica en Red en Bioingeniería, Biomateriales y Nanomedicina (CIBER-BBN), Madrid, Spain

J. Puigmartí-Luis
Departament de Ciència de Materials i Química Física, Institut de Química Teòrica i Computacional, Universitat de Barcelona, Barcelona 08028, Spain
Institució Catalana de Recerca i Estudis Avançats (ICREA); Pg. Lluís Companys 23, Barcelona 08010, Spain

X.-Z. Chen
International Institute for Intelligent Nanorobots and Nanosystems, Institute of Optoelectronics, State Key Laboratory of Photovoltaic Science and Technology, Shanghai Frontiers Science Research Base of Intelligent Optoelectronics and Perception, Fudan University, 200433 Shanghai, People's Republic of China

M. Kim
Department of Robotics and Mechatronics Engineering, DGIST, 42988, Daegu, Republic of Korea
E-mail: minkim@dgist.ac.kr

Supporting note 1: Optimization of the mesh geometry

Considering manufacturability of the wet-etching process, structural stability, and magnetoelectric stimulation efficiency, the mesh geometry was experimentally optimized. The optimization began with initial stripe widths of 1-4 μm and spacings of 1-20 μm. Following preliminary design studies across these parameter ranges (e.g., 1.5 μm stripe width with 13 μm spacing in one sample shown in Fig. S1), two types of 10×10 mm^2 lithographic masks were designed for systematic comparison (Fig. S2a-b). The mask in Fig. S2a focuses on exploring stripe width and spacing (with the number of submeshes fixed at five per row or column; i.e., the area is 370×370 μm^2.), whereas the mask in Fig. S2b focuses on refined stripe-width and spacing ranges as well as variations in the number of submeshes. Selected mesh designs were patterned on a single BTO/CFO thin film to compare the release behavior (via wet etching) and the structural robustness of the mesh. In addition to parameter optimization, two geometries, such as a regular square grid (Fig. S2c) and a staggered square grid (Fig. S2d), were also explored.

Figures S3-S5 present the visual inspection results corresponding to the mask design in Fig. S2a, showing the condition of meshes after wet etching. While an etching duration of 38 h was sufficient to fully release the geometry with 1.5 μm stripe width and 13 μm spacing (Fig. S1), it was insufficient to release any structures with stripe widths >2 μm (Fig. S3). After approximately 90 h of etching, most structures with stripe widths ≤3 μm were fully released, whereas the 4-μm designs remained only partially released (Figs. S4 and S5). We determined that the minimum etching time required for complete release is approximately 48 h for a 2 μm stripe width and 72 h for a 3 μm stripe width. Although narrower stripe widths require shorter etching times and show less breakage, they exhibit reduced ME material coverage. Therefore, stripe widths of 2-3 μm with spacings of 5-9 μm were identified as optimal for maintaining both robustness and sufficient ME material coverage.

To mitigate the risk of breakage, the mesh was divided into smaller submeshes. This approach confines potential damage to a limited area and preserves most of the active material even when local fractures occur. As shown in Fig. S6 (corresponding to the mask design in Fig. S2b), the sample surface was divided into varying numbers of submeshes (40 or 60), with each submesh measuring approximately 200×200 μm^2 or 130×130 μm^2, separated by ~10 μm spacing. The results indicate that smaller submeshes and a larger number of submeshes improve material coverage and structural reliability.

In conclusion, the mesh design parameters are set to a stripe width of 2-3 μm, a spacing of 5 to 9 μm, and a submesh size of approximately 150×150 μm^2. We selected the regular grid geometry as the final design, as meshes with this geometry consistently exhibited high robustness after release, whereas those with the staggered grid geometry were prone to near-complete breakage. This submesh design was homogeneously patterned over a square area of ≈ 8.5×8.5 mm^2 and positioned at the center of the 10×10 mm^2 mask (Fig. S7).

Through a two-step photolithography process, the photoresist edge beads were first removed, and then the mesh was patterned using the photomask with the optimized design. In the first step, the edge beads were etched away by a preliminary exposure of the sample edges, followed by resist stripping. A second photolithography step was then performed using the mask with the final geometry. This approach ensured good contact between the photolithographic mask and the sample surface, thereby resulting in excellent mask-to-sample transfer fidelity, as shown in Fig S7c and d. After full manufacturing process, the final clamped/freestanding/PDMS-transferred meshes were achieved (Fig. S8).

To complement the experimental optimization of the ME mesh design, finite element analysis (FEA) was conducted using COMSOL Multiphysics. The simulation model incorporated the intrinsic material properties of BTO and CFO and considered the interfacial strain arising from their lattice parameter mismatch. By varying the interfacial strain, the mechanical behavior of bilayer BTO/CFO meshes was evaluated under different geometric configurations. Structural parameters, including stripe width (1, 2, and 3 μm), spacing (4, 8, and 12 μm), and the number of patterns (2×2, 4×4, and 6×6), were systematically varied. The key mechanical properties of interest were the maximum deflection and the stress concentration at the corners, as both are critical factors that can lead to structural damage during fabrication and processing.

Figure S9 presents an example of the simulated out-of-plane (z-axis) deflection. The summarized results (Fig. S10) show that the maximum deflection increased with larger spacing and smaller stripe width. This observation is consistent with analytical expectations: narrower stripes are mechanically more flexible (lower stiffness), while wider spacing promotes greater out-of-plane deformation under identical shear strain. Increasing the number of mesh patterns (representing repeated unit designs) led to a slight increase in total deflection due to the accumulation of elastic deformation across the mesh.

For the stress concentration analysis (example shown in Fig. S11), the average von Mises stress at the mesh corners was evaluated. Figure S12 summarizes how the average stress varied with design parameters. As the stripe width increased, the average stress also increased; however, the stress levels remained below the material failure threshold up to a width of 3 μm.[1] It should be noted that this simulation employed bulk BTO and CFO material properties, which may deviate from those of nanoscale thin films, where lower Young's modulus and higher failure strength are often observed. Thus, more detailed material property modeling would improve the accuracy of the simulation.

Overall, the simulation results support the experimentally optimized design parameters of a stripe width of 2-3 μm and a spacing of 5-9 μm. Notably, simulations of 1 μm stripe width also showed promising mechanical behavior; however, this configuration was excluded experimentally to ensure a higher material packing density and enhanced electrical energy generation. The stripe width can be tuned within the range of 1-3 μm depending on the specific device requirements and target applications. It should also be noted that wider stripes, while providing greater rigidity, can induce higher residual tension leading to potential device failures, whereas narrower stripes reduce internal stress but at the cost of lower structural stiffness. Therefore, a balanced design must be achieved to ensure both mechanical reliability and functional efficiency of the ME mesh.

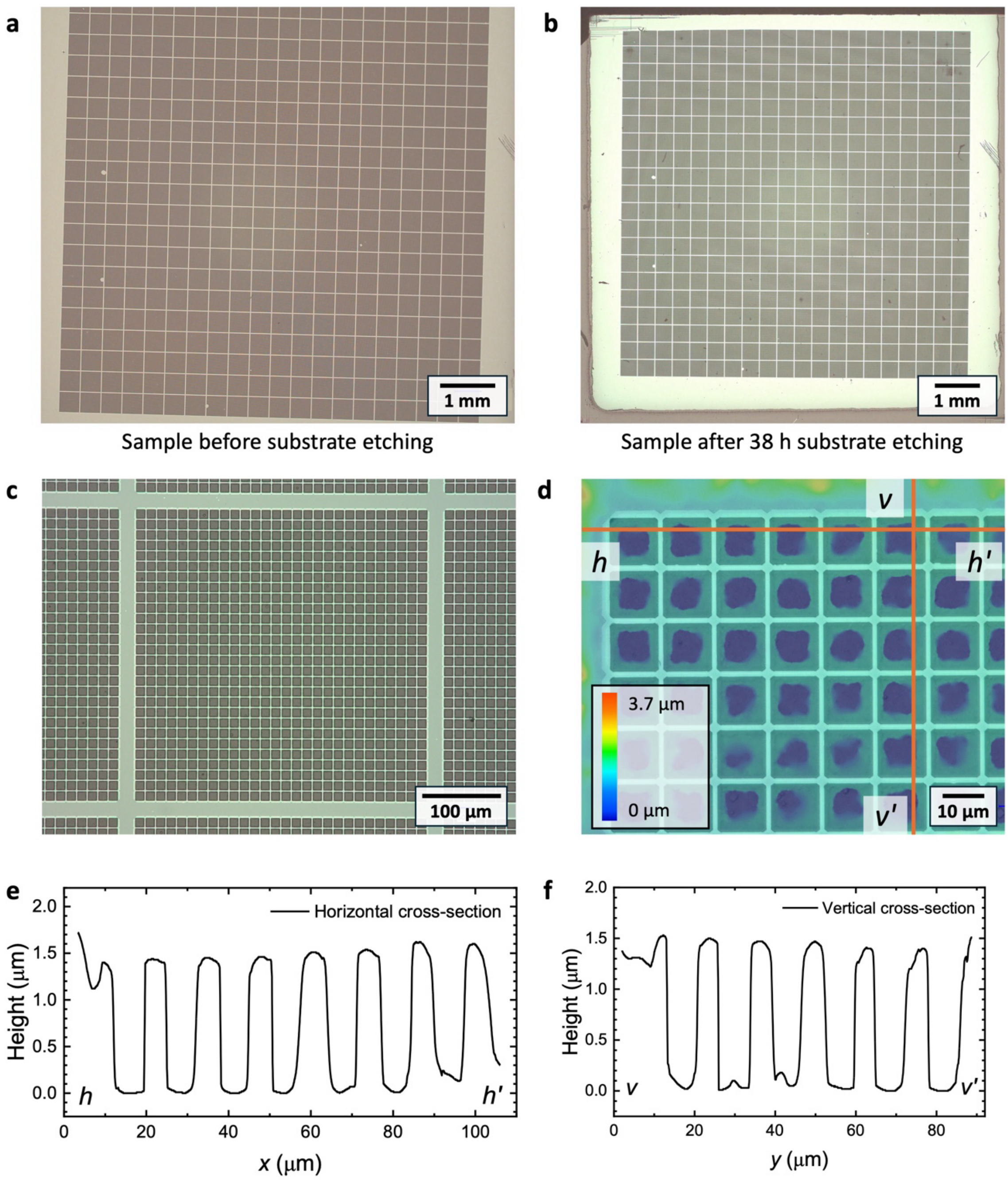

Figure S1: Mesh geometry consisting of stripes of 1.5 µm stripe width with 13 µm spacing. (a) Optical image before substrate etching. (b) Optical image after 38 h etching of the MgO substrate. (c) Selected magnified views from (b). (d) Height map image near a submesh corner, highlighting the homogeneity of the release process. (e-f) Cross-sectional profiles corresponding to (d). The etched lateral distance is ≈2.4 µm and the etched depth is ≈1.5 µm. While this geometry exhibits excellent release and structural robustness, it compromises ME stimulation efficiency because the remaining ME material after patterning is low (≈22%).

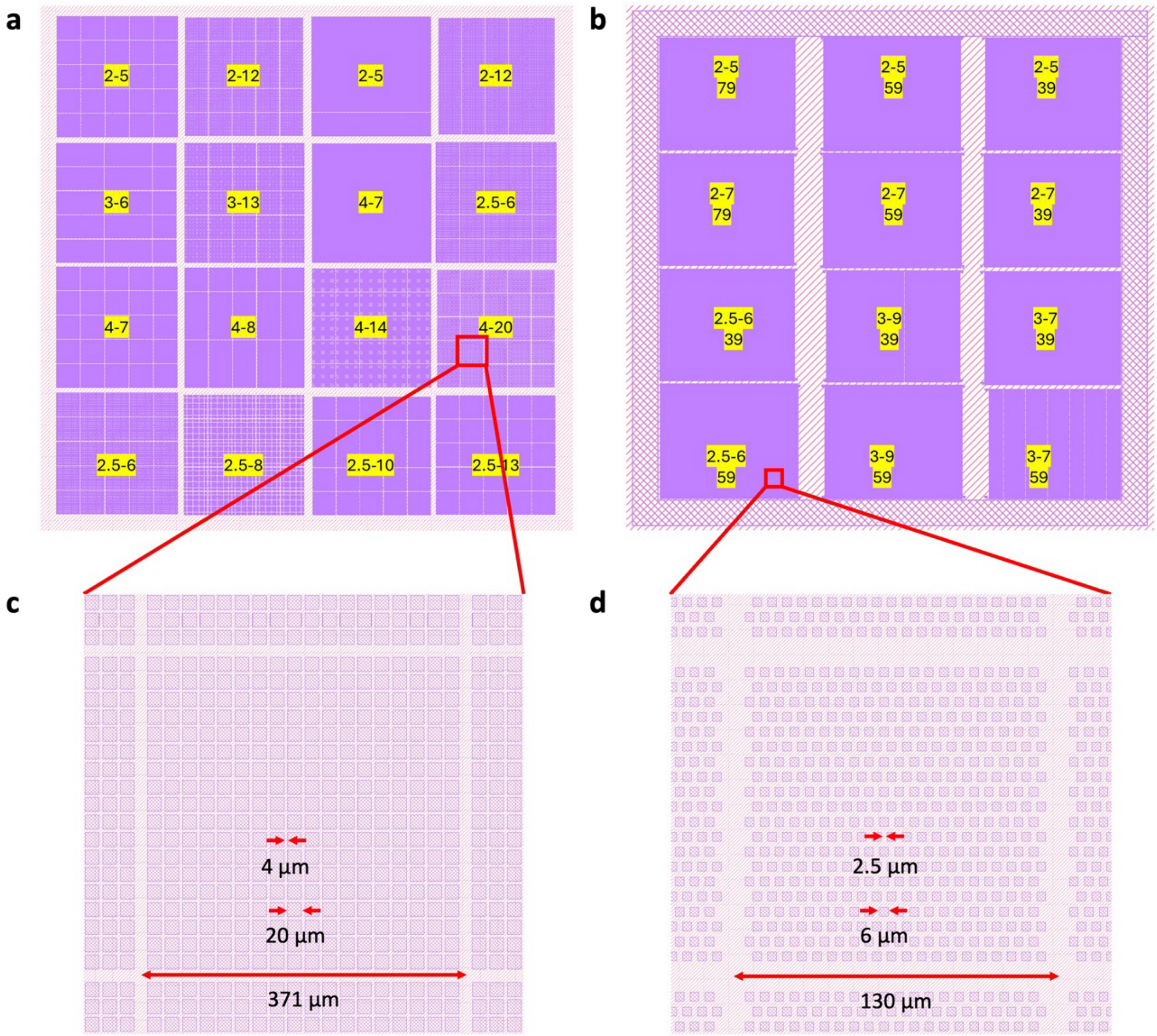

Figure S2: KLayout designs for mesh patterning. The pink regions correspond to the remaining BTO/CFO material forming the mesh stripes, while the purple areas indicate etched openings. The "m-n" notation in the insets refers to the stripe width m and spacing n (in µm). (a) Multiple mesh designs exploring stripe widths of 2-4 µm and spacings of 2-20 µm. The number of submeshes per row (or column) is 5. (b) Multiple mesh designs exploring stripe widths of 2-3 µm with spacings of 5-9 µm, along with variations in the number of submeshes. The numbers 39, 59, and 79 indicate the separations dividing the submeshes, corresponding to 40, 60, and 80 submeshes fabricated per row (or column), respectively. (c) Magnified view of a regular square grid. (d) Magnified view of a staggered square grid. Both examples illustrate how the stripe width and spacing are implemented in the mesh pattern.

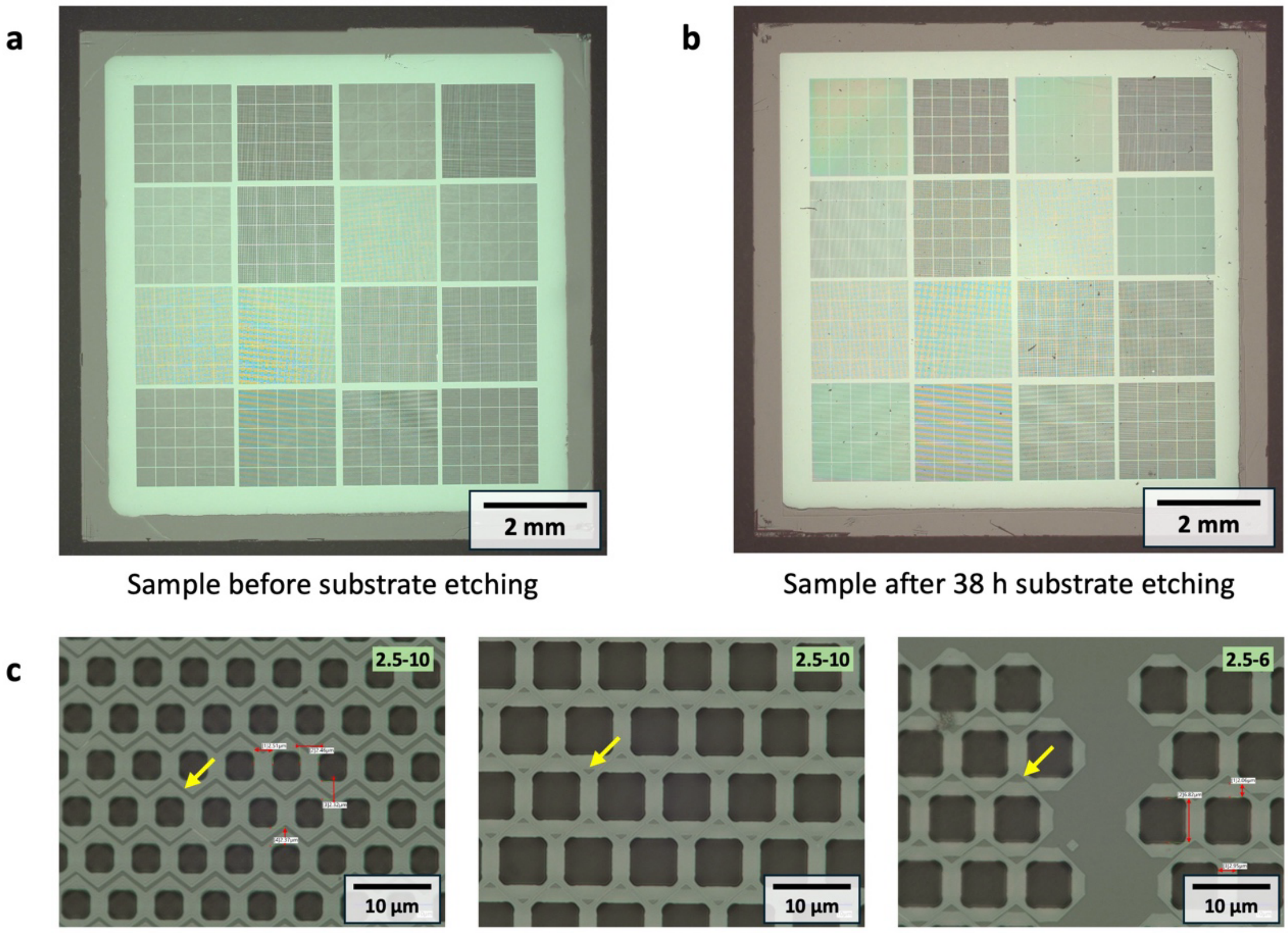

Figure S3: Time-dependent substrate etching results (38 h). The mesh design corresponds to that shown in Fig. S2a. (a) Optical image before substrate etching. (b) Optical image after 38 h etching of the MgO substrate. (c) Selected magnified views from (b). The "m-n" notation in the insets refers to the stripe width m and spacing n (in µm). Yellow arrows highlight regions of incomplete release. The dark regions, corresponding to clamped films, indicate that the mesh remains only partially released and is not fully freestanding.

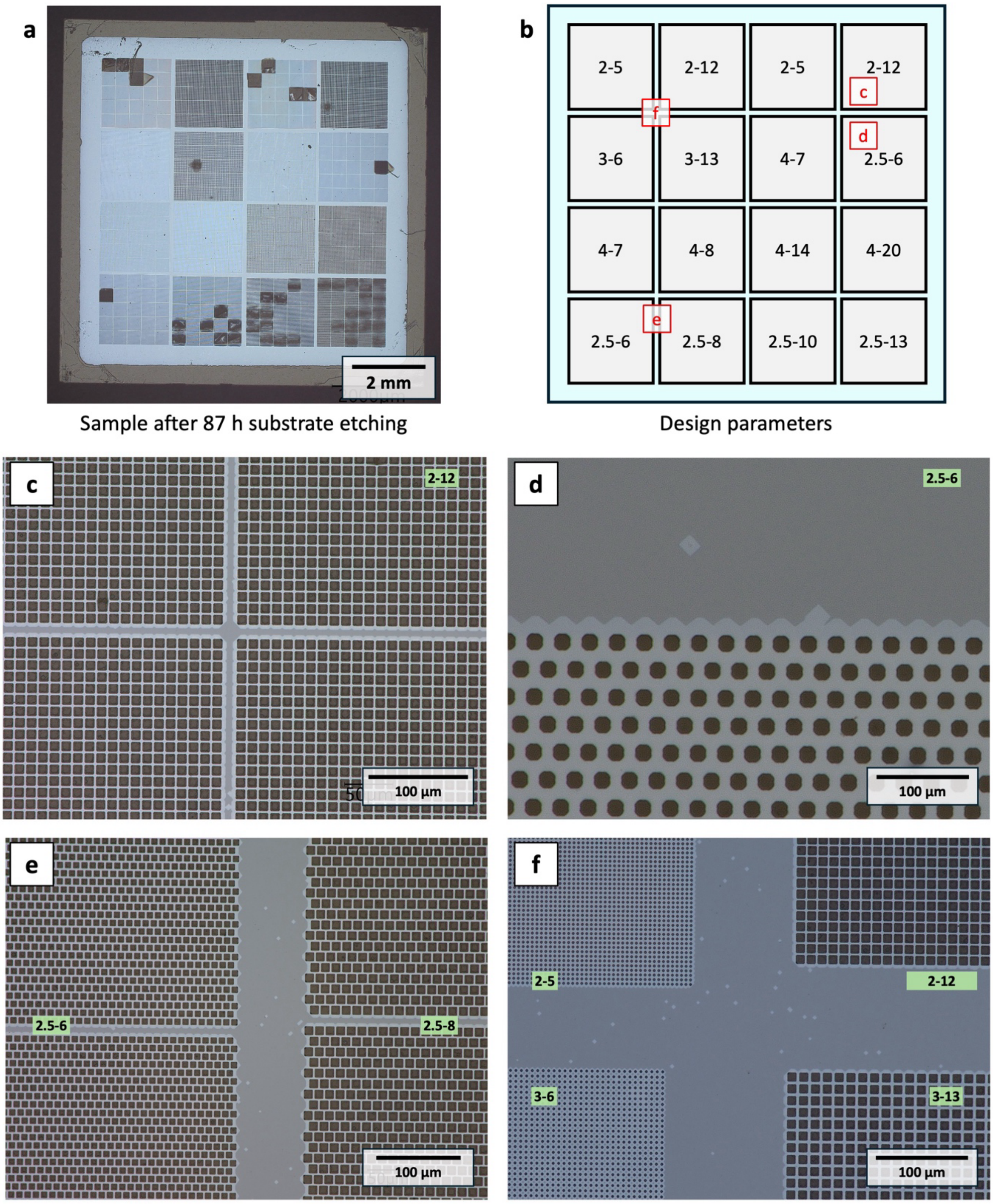

Figure S4: Time-dependent substrate etching results (87 h). (a) Optical image after 87 h etching of the MgO substrate. (b) Corresponding design parameters. (c-f) Selected magnified views from (a-b). The "m-n" notation in the insets refers to the stripe width m and spacing n (in µm). Mesh designs with stripe widths ranging from 2 to 3 µm show fully released, freestanding structures. Although some narrower stripe-width designs became fully released and even showed instances of fracture, none of the 4-µm stripe-width designs were fully released. Yellow arrows highlight regions of incomplete release.

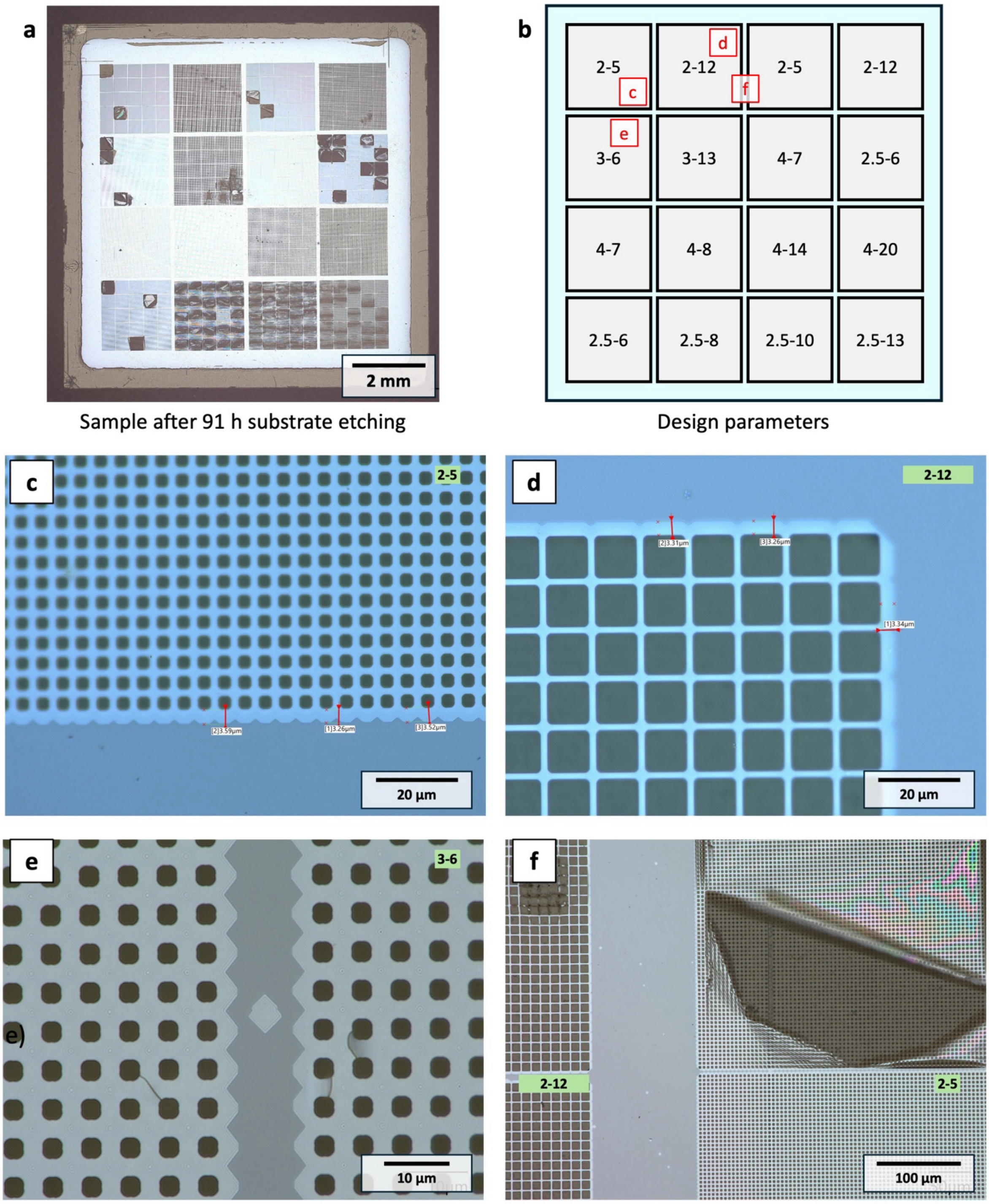

Figure S5: Time-dependent substrate etching results (91 h). (a) Optical image after 91 h etching of the MgO substrate. (b) Corresponding design parameters. (c-f) Selected magnified views from (a-b). The "m-n" notation in the insets refers to the stripe width m and spacing n (in µm). Mesh designs with stripe widths ranging from 2 to 3 µm show fully released, freestanding structures. Although some fractured meshes are observed in (f), none of the 4-µm stripe-width designs were fully released.

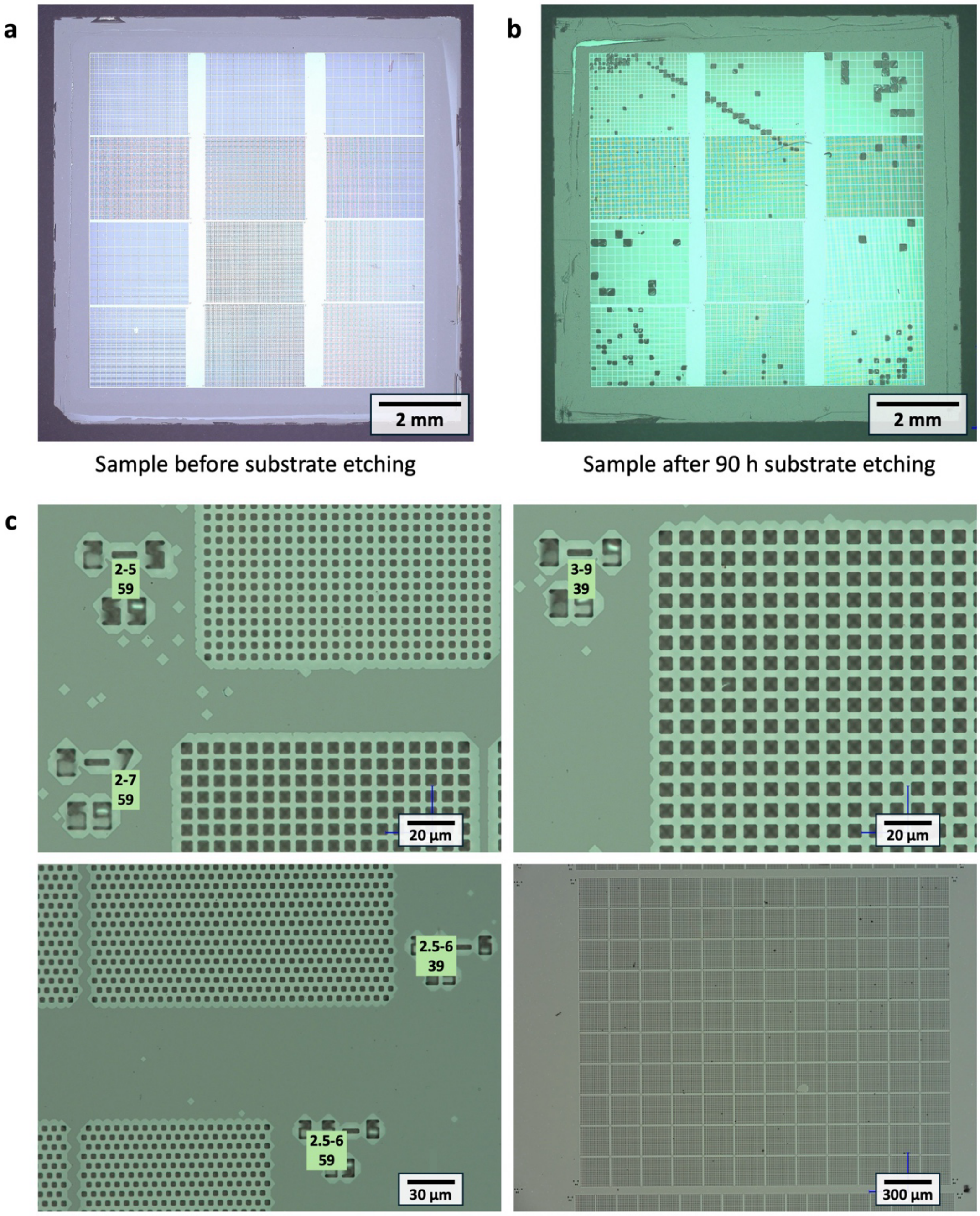

Figure S6: Time-dependent substrate etching results (90 h). The mesh design corresponds to that shown in Fig. S2b. (a) Optical image before substrate etching. (b) Optical image after 90 h etching of the MgO substrate. (c) Selected magnified views from (b). The "m-n" notation in the insets refers to the stripe width m and spacing n (in µm). The numbers 39 and 59 indicate the separations dividing the submeshes; that is, 40 and 60 submeshes per row (or column) were fabricated, respectively.

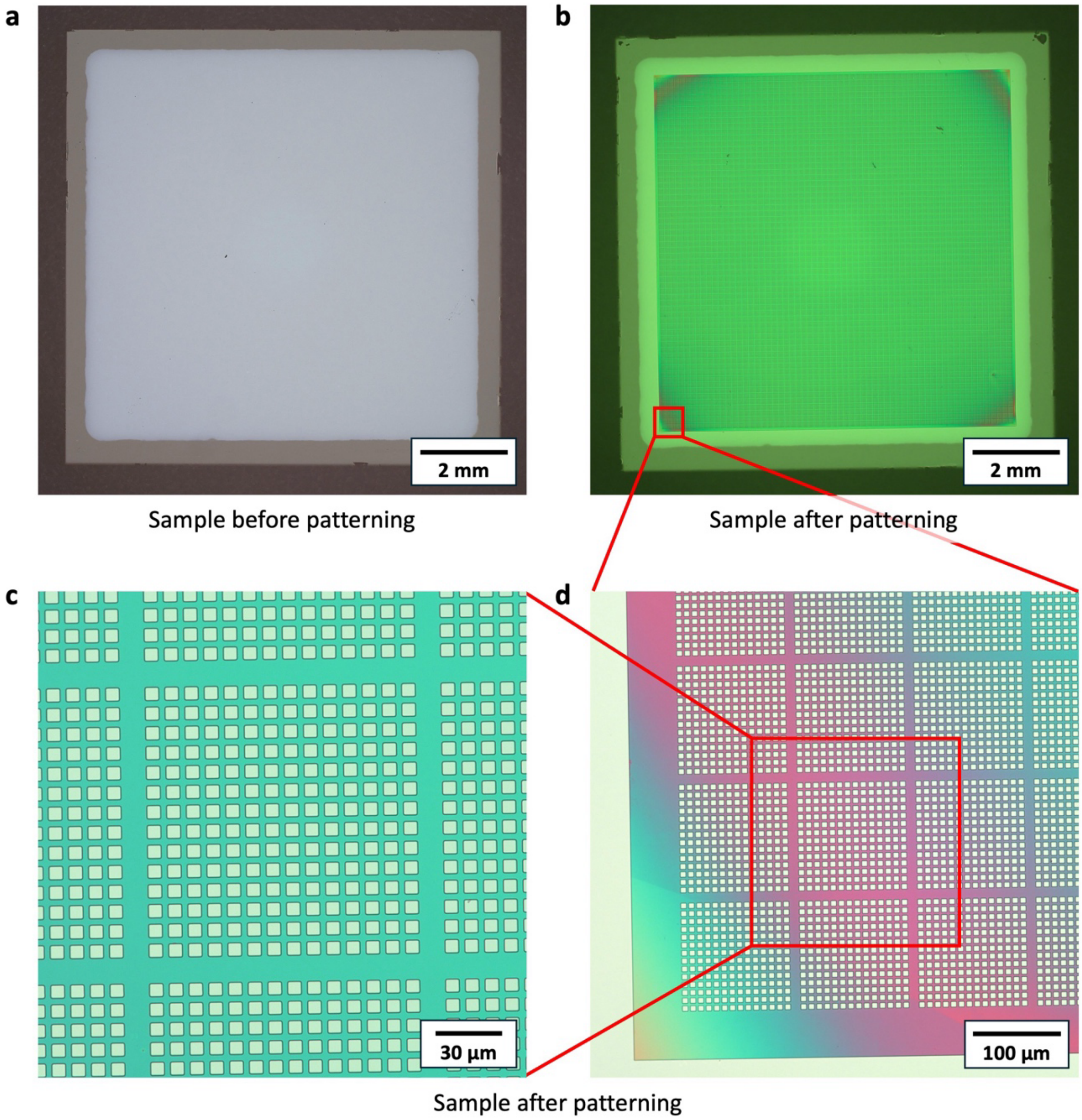

Figure S7: Photolithography step for mesh patterning. (a) As-deposited BTO/CFO bilayer. (b) Bilayer after photoresist patterning, prior to physical etching. (c-d) Magnified views highlighting the high fidelity of the pattern transfer.

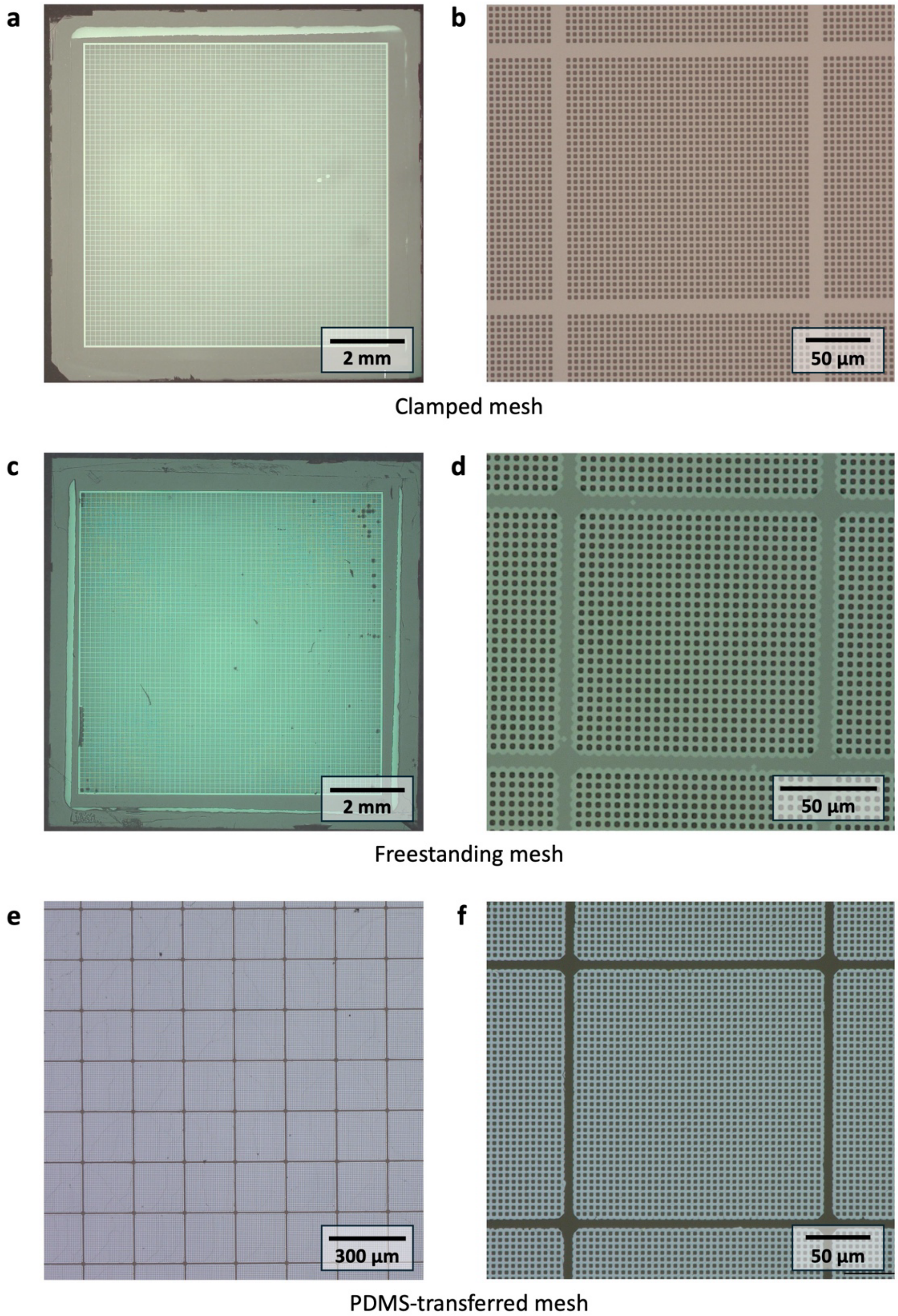

Figure S8: Optical images of BTO/CFO meshes in each boundary condition. (a-b) Substrate-clamped mesh. (c-d) Freestanding mesh. (e-f) PDMS-transferred mesh. The images on the left show a zoomed-out view of the sample, while the images on the right provide a magnified view of a single submesh region.

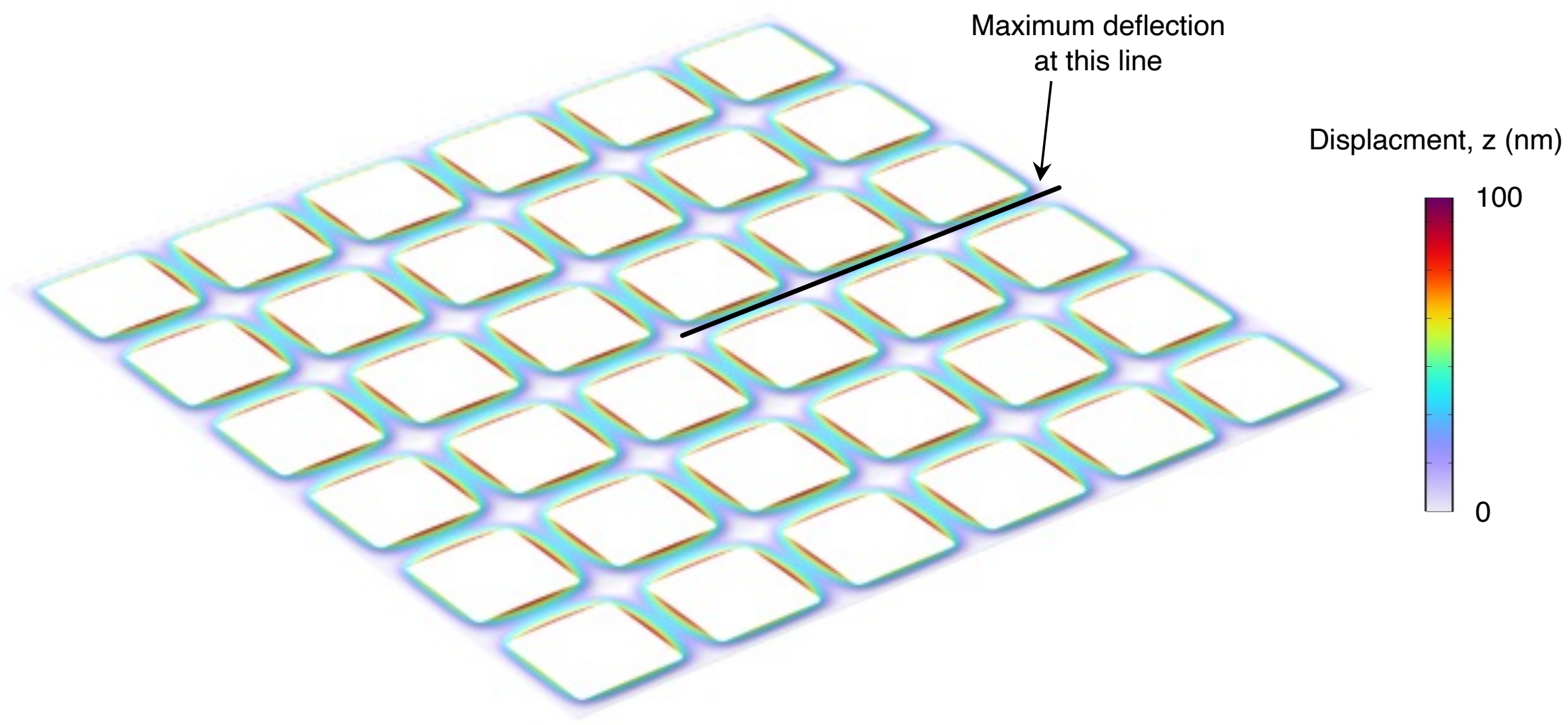

Figure S9: Out-of-plane displacement of the ME mesh with a 2 μm stripe width, 8 μm spacing, and a 6×6 pattern array.

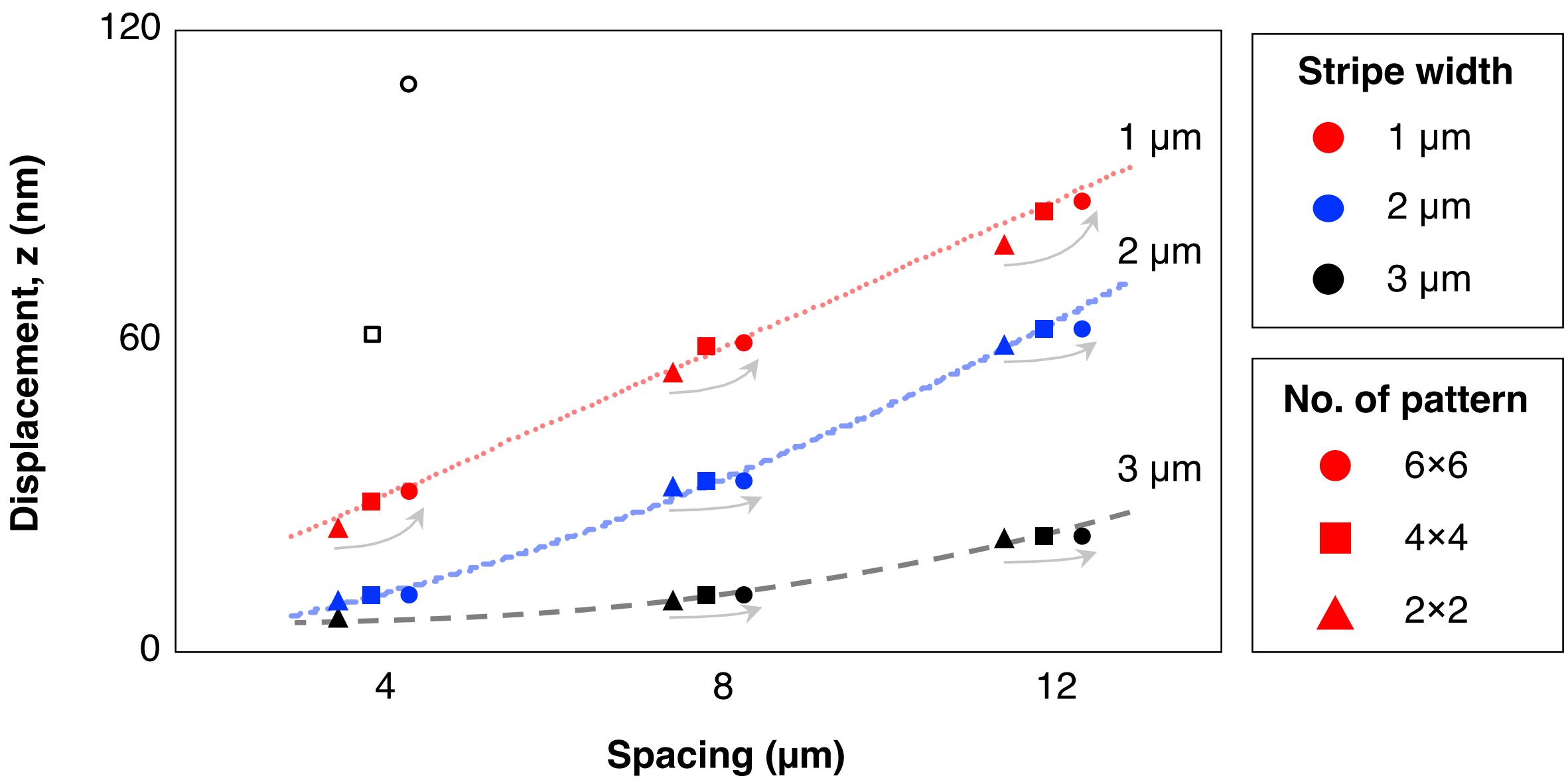

Figure S10: Simulated maximum displacement along the line indicated in Figure S9 of the ME mesh for different stripe widths, spacing, and number of patterns.

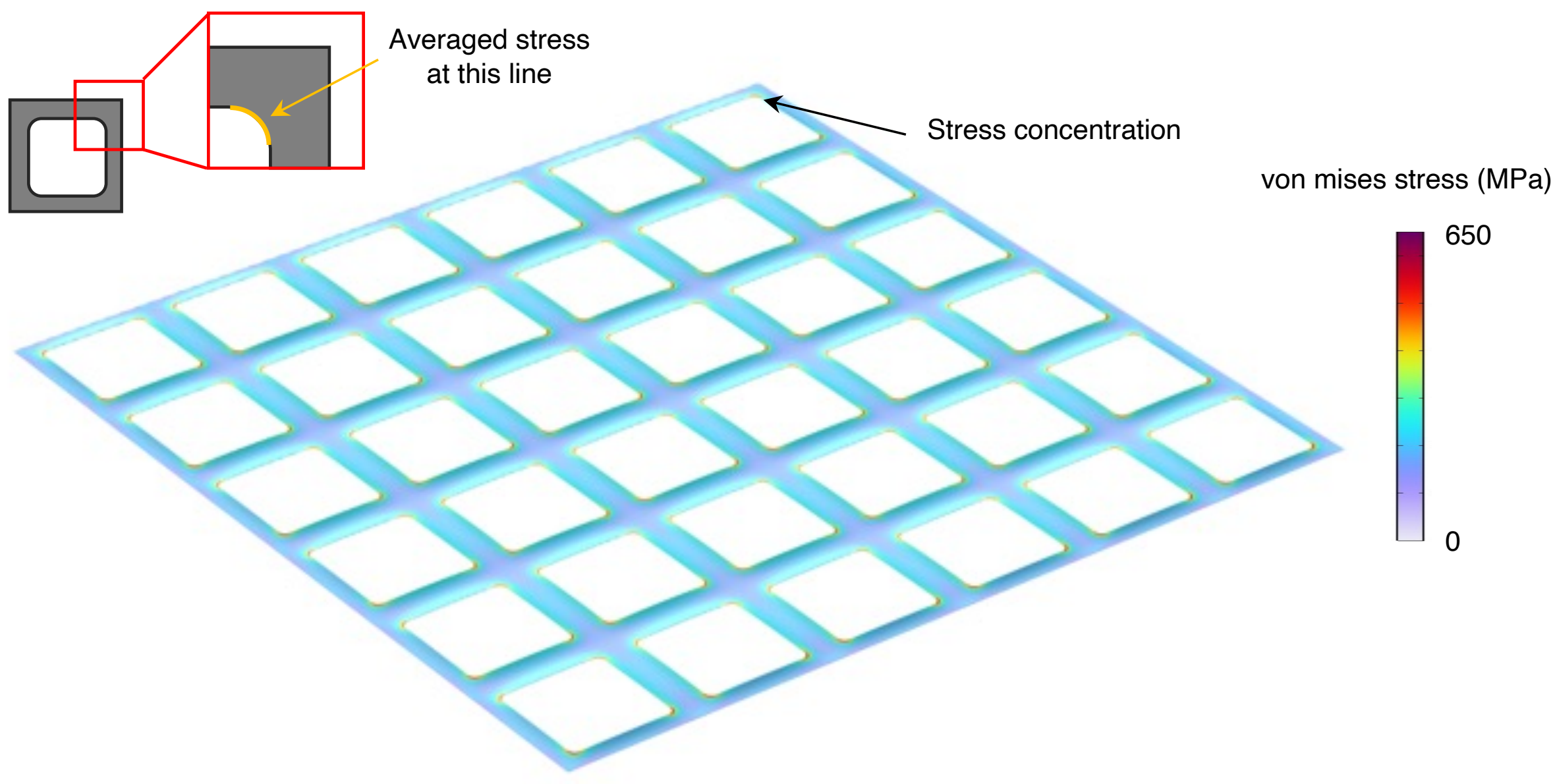

Figure S11: Stress distribution of the ME mesh with a 2 μm stripe width, 8 μm spacing, and a 6×6 pattern array.

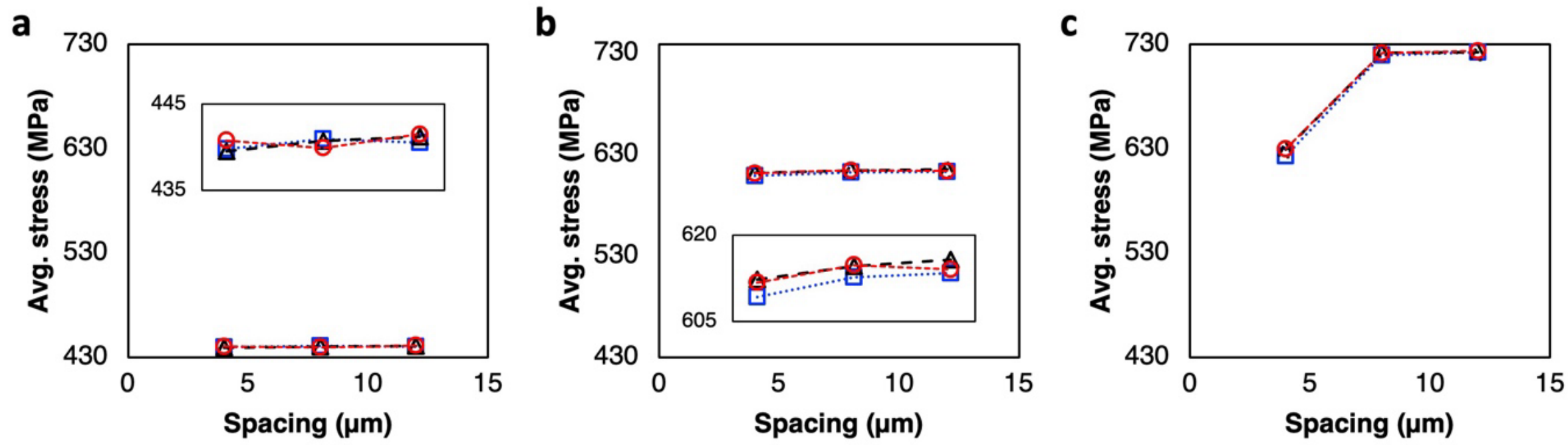

Figure S12: Simulated stress concentration at the corners (averaged as shown in Fig. S11) of the ME mesh for different stripe widths: (a) 1 μm, (b) 2 μm, and (c) 3 μm.

Supporting note 2: Structural characterization

Structural characterization was performed by X-ray diffraction. Fig. S13a (resp. Fig. S13b) shows the θ-2θ scan around the 002 MgO reflection for a clamped BTO/CFO bilayer (resp. a freestanding BTO/CFO mesh). The CFO 004 reflection ($2\theta \approx 43.2°$) is present as a small shoulder on the right side of the MgO peak ($2\theta = 42.915°$) because of the closely matching cell parameters between MgO ($a_{bulk} = 4.210$ Å) and CFO ($a_{bulk} = 4.190$ Å), while the BTO 002 peak is located at $2\theta \approx 45.4°$. Interestingly, the CFO and BTO c-axis parameters of both clamped and freestanding samples are identical with $c_{CFO} = 4.186$ Å and $c_{BTO} = 3.996$ Å, and Laue fringes of CFO are visible in both samples. This observation indicates that the release process does not degrade the BTO/CFO bilayer, which retains its high crystalline quality, constant thickness (15 nm/15 nm), and strain state. Moreover, the c-axis of CFO is slightly smaller than the bulk one, hinting at a fully tensile strained film onto MgO, while the c-axis of BTO indicates of prevalence of in-plane a/b domains, also consistent with the high tensile strain imposed by the substrate. These observations are consistent with our previous results on similar BTO/CFO bilayers.[2]

The long-range θ-2θ scan of the freestanding mesh (Fig. S13c) confirms the above-mentioned observations with the presence of 004 and 008 CFO peaks at $2\theta \approx 43.2°$ and $2\theta \approx 94.8°$, respectively, and the presence of 001 and 002 BTO peaks at $2\theta \approx 22.25°$ and $2\theta \approx 45.4°$, respectively, while 003 BTO (≈ 70.6°) and 004 BTO (≈ 100.9°) are nearly not visible due to intrinsic material properties (i.e., different structure factors).

Note that, in a 50 nm thick CFO single layer, deposited to see a clear CFO peak, rocking curves have similar FWHM in CFO (≈0.02°) compared to MgO attesting of the high crystallinity of the CFO layer, also confirmed by presence of Laue fringes. A similar high crystallinity is expected in the 15 nm CFO layer underneath the 15 nm BTO layer. On the other hand, the BTO rocking curves reveal quite broader rocking curve without significant change between the three mesh configurations (FWHM ≈ 1.7° for clamped and freestanding configurations and FWHM ≈ 1.9° for the PDMS-transferred one; cf. Fig. S13d), which can be explained by the high mismatch between CFO and BTO forcing BTO to relax after few unit cells (as confirmed by TEM; cf. main text). Such strain relaxation is probably accommodated by the formation of defects such as dislocations or point defects.

The atomic force microscopy (AFM) data of the as-deposited BTO/CFO bilayer (Fig. S14) reveals an atomically flat surface with root-mean-square (RMS) roughness of ≈0.4 nm.

Fig. S15 (resp. Fig. S16) shows the surface morphology of a clamped BTO/CFO mesh onto MgO (resp. a PDMS-transferred mesh). From the cross-sections of the PDMS-transferred mesh, the thickness of BTO/CFO (≈30 nm) is observed, which confirms both preferential etching of the MgO substrate alone and preservation of the film integrity along the whole process. Therefore, the film has also been perfectly transferred to the host PDMS substrate (despite the presence of MgO residues at the stripe intersections), thus discarding film degradation provoked by the different etching steps.

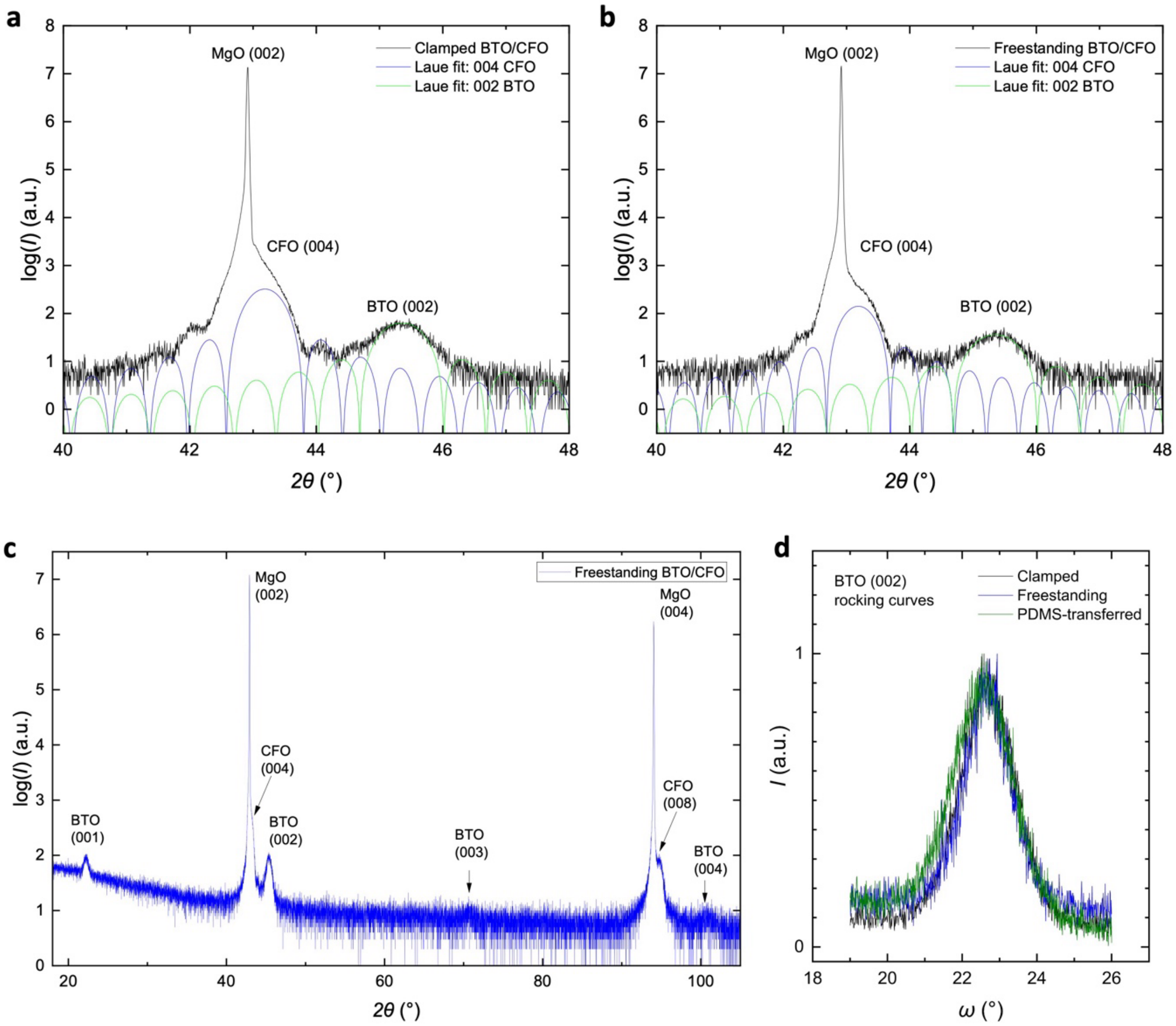

Figure S13: X-ray diffraction data. (a) *θ-2θ* scan around 002 MgO reflection of an as-deposited BTO/CFO bilayer. (b) *θ-2θ* scan around 002 MgO reflection of a freestanding BTO/CFO mesh. (c) Long range *θ-2θ* scan of a freestanding BTO/CFO mesh. (d) *ω* scans (i.e., rocking curves) of the 002 BTO reflection in the three mesh configurations.

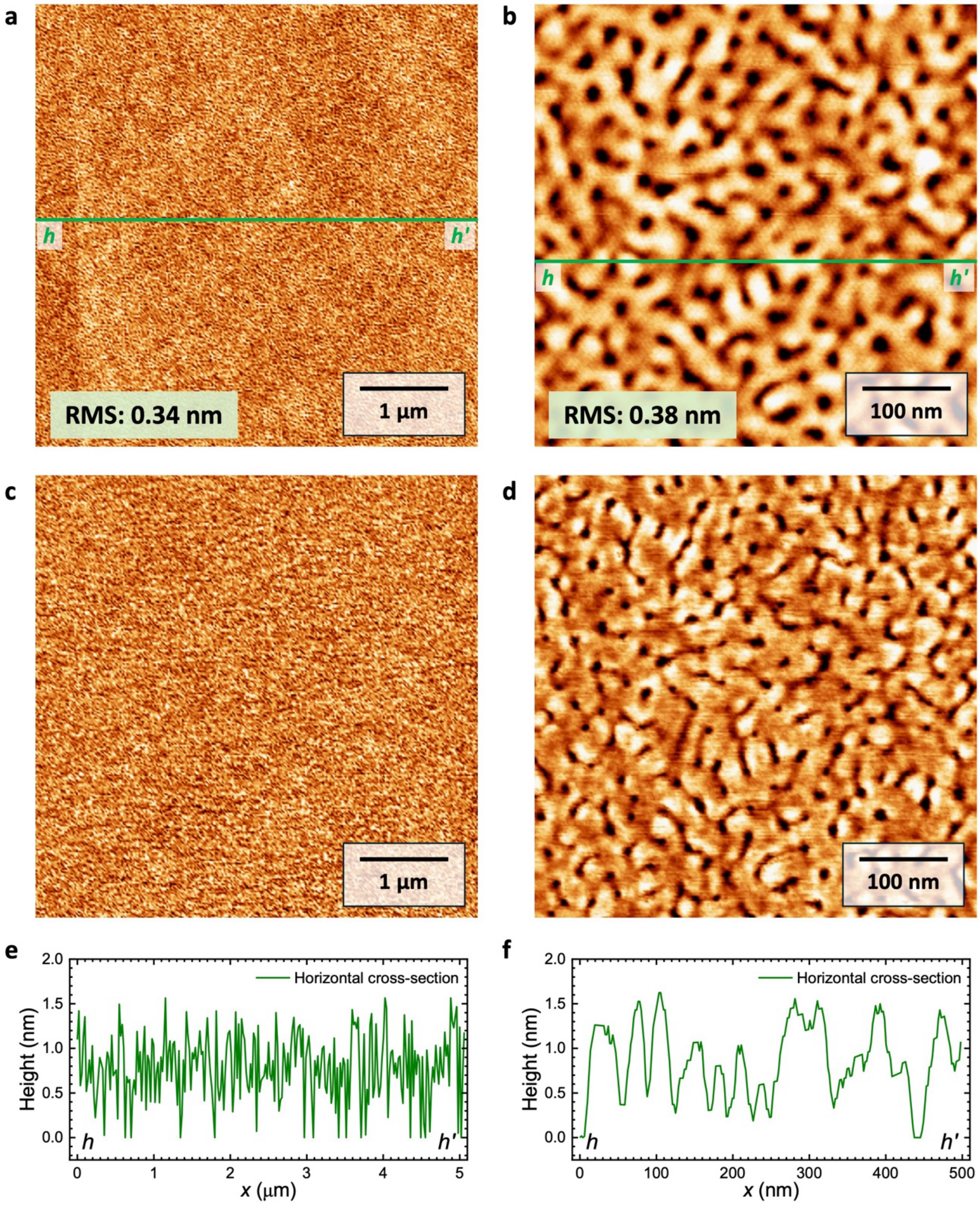

Figure S14: Atomic force microscopic data of a clamped BTO/CFO bilayer after deposition. (a) Height profile over a 5×5 μm^2 surface. (b) Magnified 500×500 nm^2 image of the same region. The extracted surface RMS roughness is 0.36±0.02 nm for both magnifications. (c-d) Respective phase images. (e-f) Extracted horizontal cross-sections for both magnifications, showing peak-to-valley height of ≈1.5 nm.

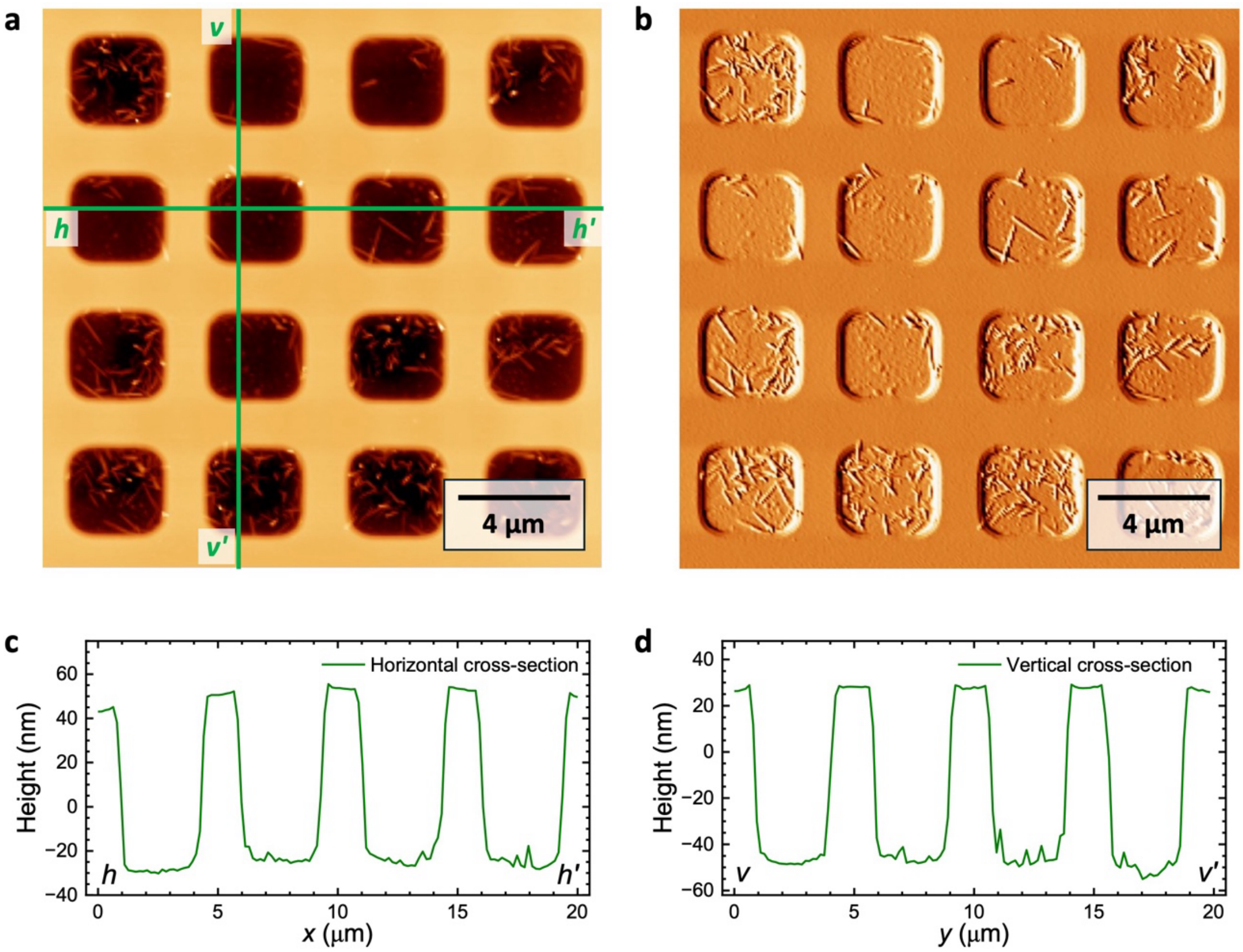

Figure S15: Atomic force microscopic data of a clamped BTO/CFO mesh on MgO substrate. (a) Height profile. (b) Phase image showing more defined edges. (c-d) Profile of horizontal and vertical cross-section taken from the height profile image. In both cross-sections, the peak-to-valley height is around 80 nm, highlighting the etching depth achieved by dry etching (ion-milling).

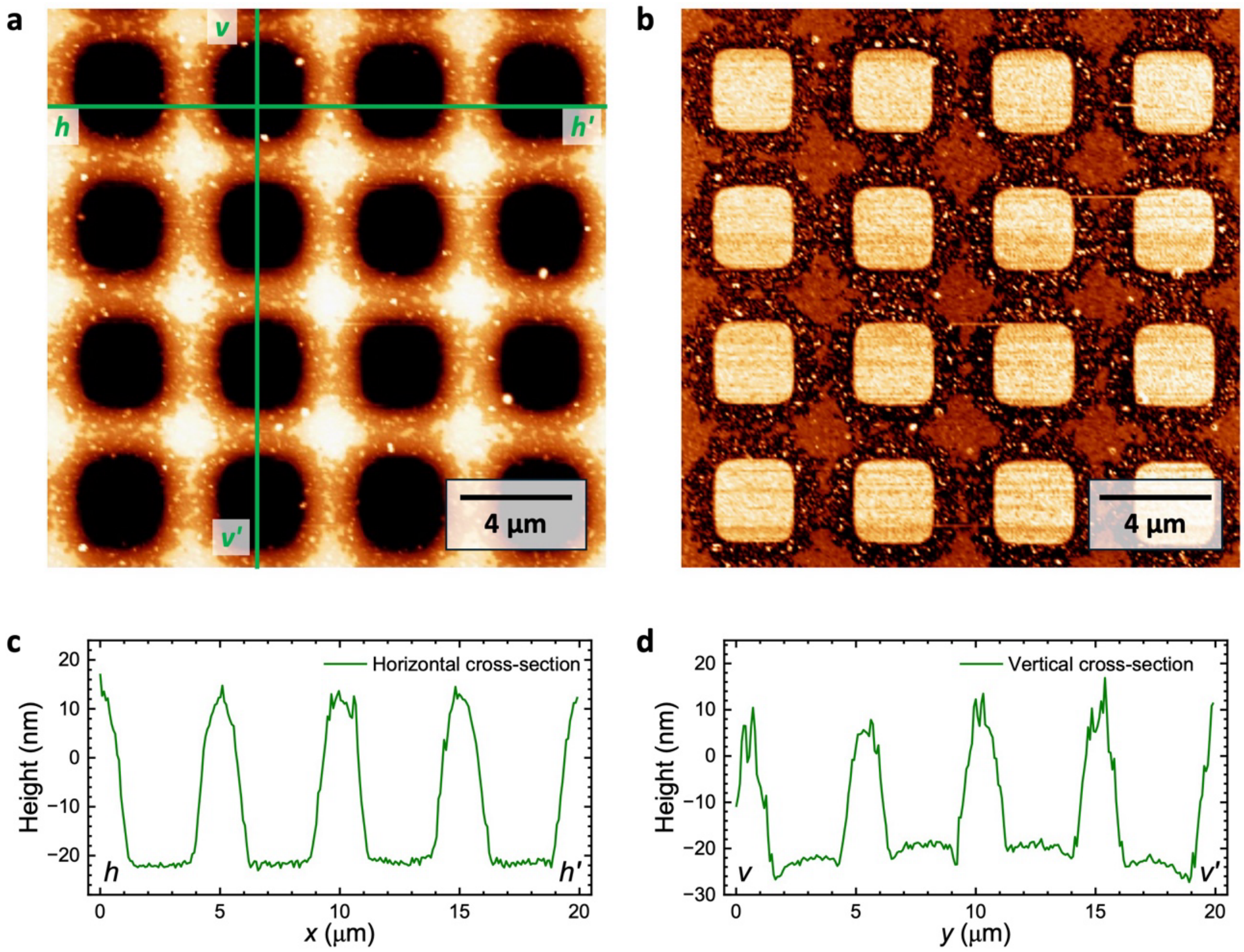

Figure S16: Atomic force microscopic data of a PDMS-transferred BTO/CFO mesh. (a) Height profile. (b) Phase image showing more defined edges. (c-d) Profile of horizontal and vertical cross-section taken from the height profile image. In both cross-sections, the mesh height is around 30 nm thus confirming that the whole bilayer quality and integrity is preserved during the process (patterning and transfer).

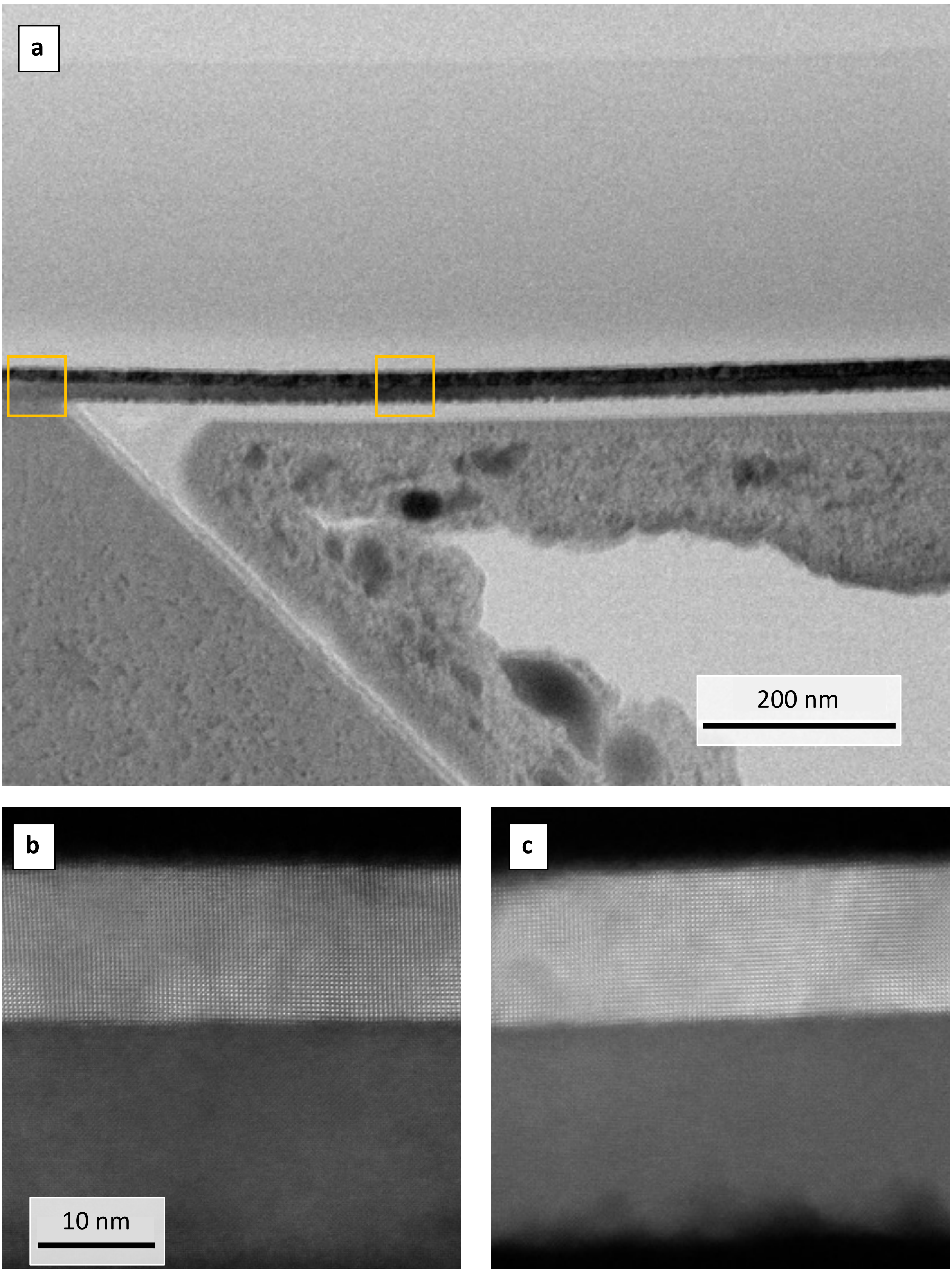

Figure S17: Cross-sectional TEM images of a freestanding mesh. (a) Low-magnification view showing the TEM lamella with a substrate-clamped mesh (left, orange square) and a freestanding mesh (right, orange square). (b) Magnified view of the clamped mesh. (c) Magnified view of the freestanding mesh.

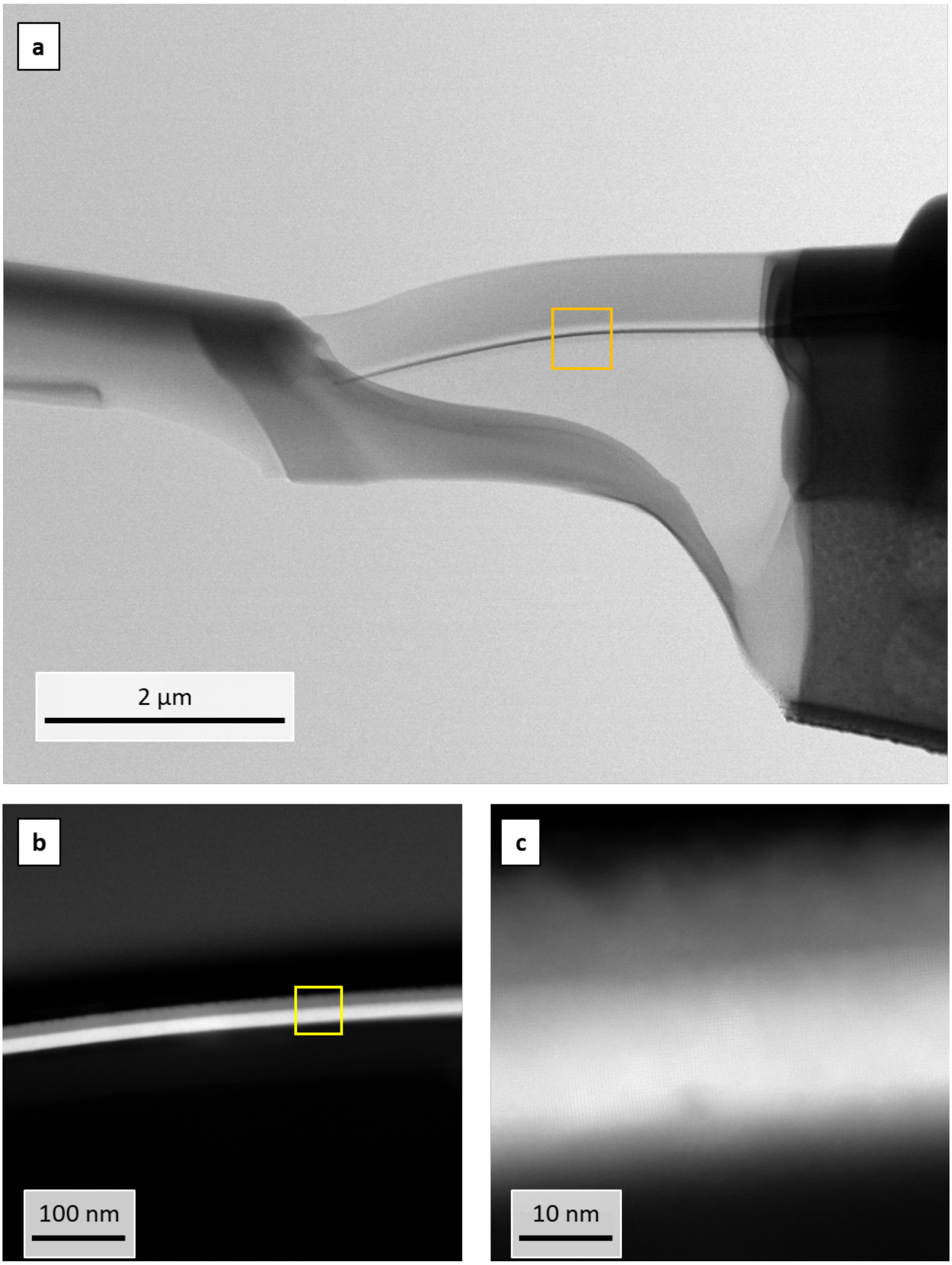

Figure S18: Cross-sectional TEM images of a PDMS-transferred mesh. (a) Low-magnification view showing the TEM lamella. (b) Higher-magnification view corresponding to the orange square in (a). (c) Magnified view corresponding to the yellow square in (b).

Supporting note 3: Complementary PFM measurements

The shift observed in the phase and amplitude measurements during the application of the magnetic field is fully recovered once the field is switched off, as illustrated in Fig. S19. This behavior demonstrates the reproducibility of the magnetoelectric effect measurements in our BTO/CFO mesh.

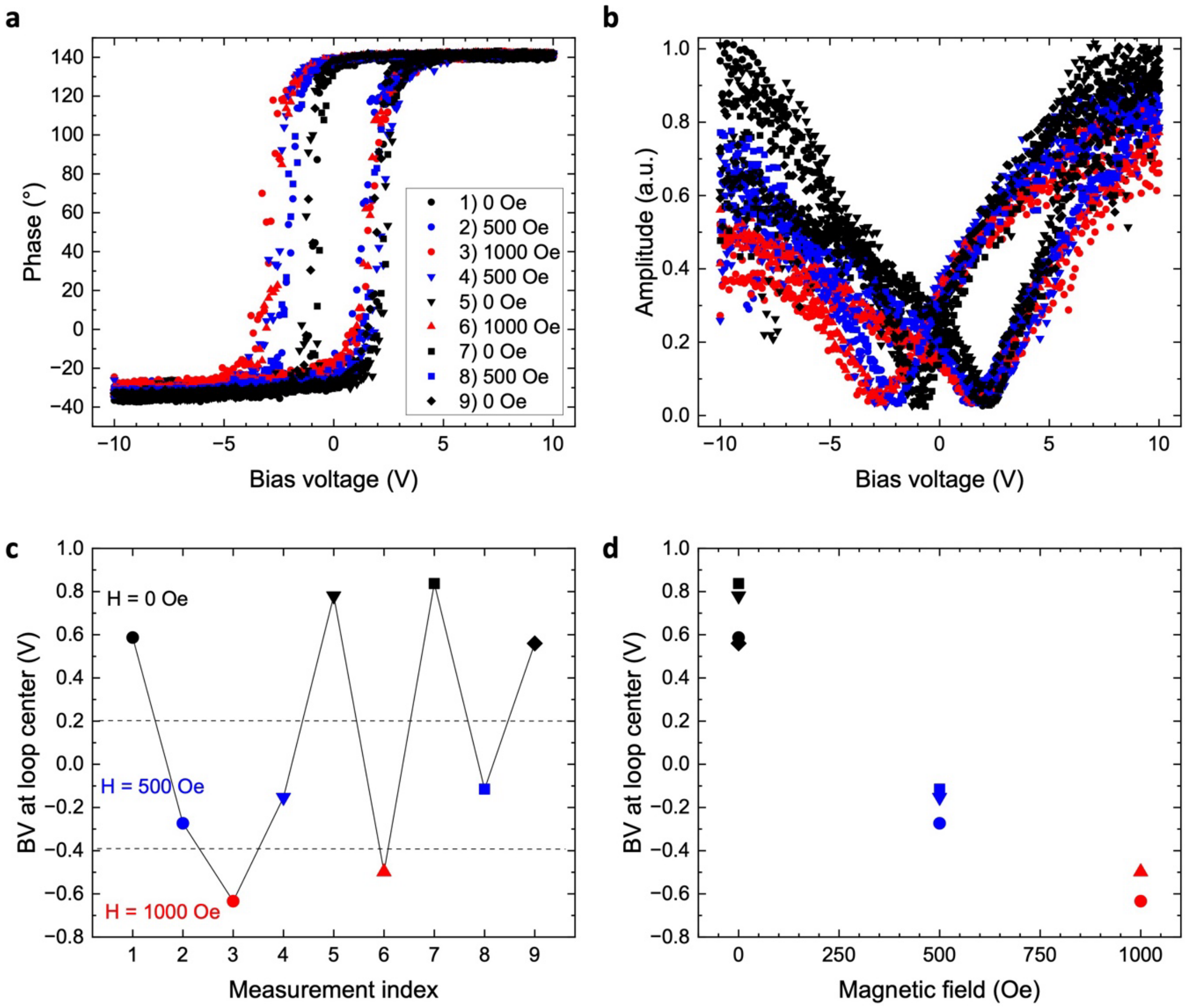

Figure S19: PFM measurements performed under different applied magnetic fields on a freestanding mesh transferred onto an Au-coated Si host substrate. (a) Phase response under different magnetic field conditions. The numbers in the legend indicate the measurement index (i.e., the experiment number) and the corresponding magnetic field strength. (b) Amplitude response under different magnetic field conditions. (c) Bias voltage at the center of the phase loop as a function of measurement index. (d) Bias voltage at the center of the phase loop as a function of applied magnetic field.

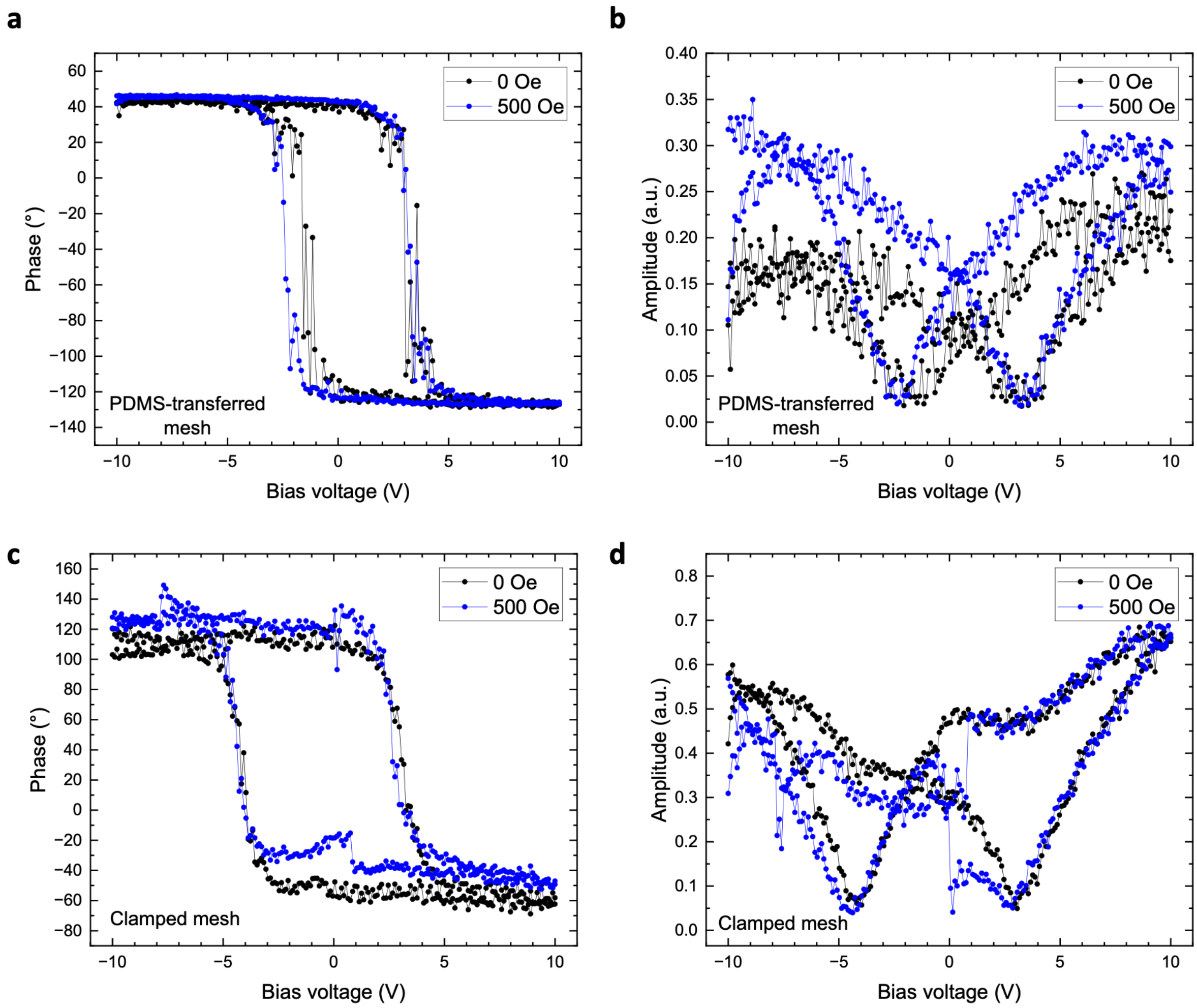

Figure S20: PFM measurements performed under different applied magnetic fields on: PDMS-transferred mesh (a-b) and clamped mesh (c-d). Left (resp. right) panels show the phase (resp. amplitude) response under different magnetic field conditions.

Supporting note 4: Cell differentiation results

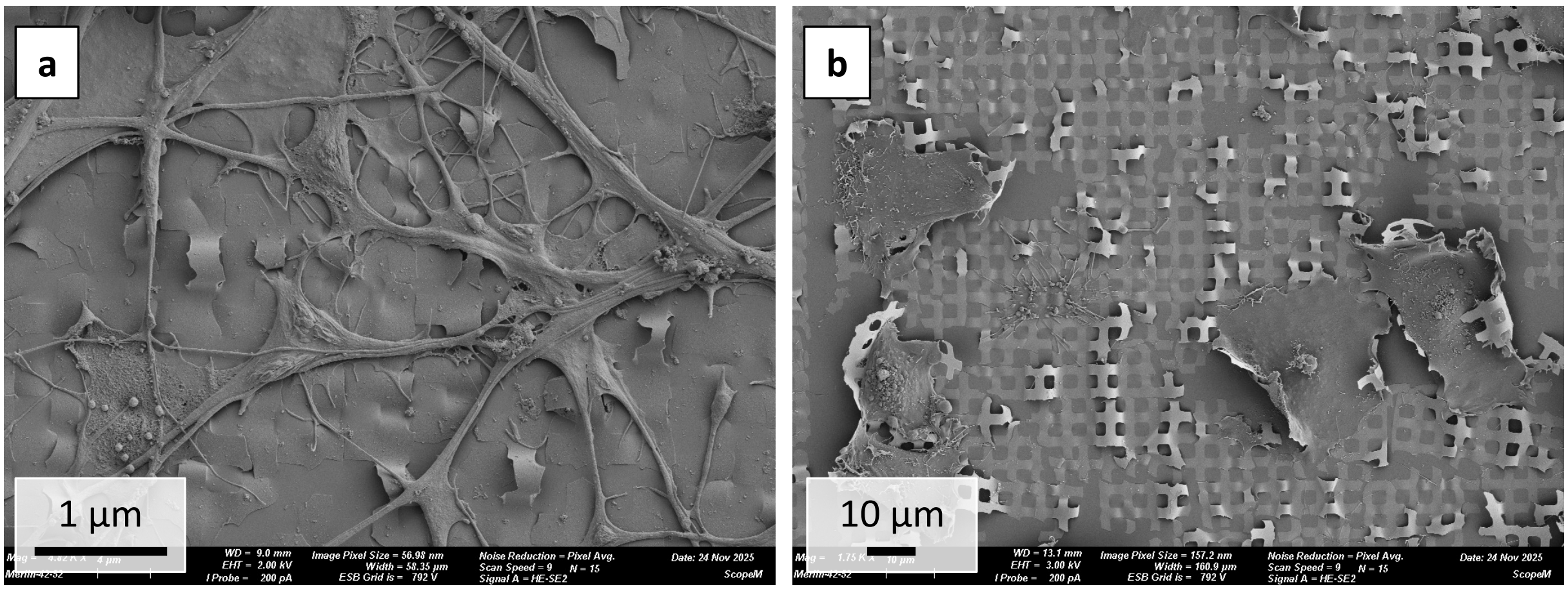

Figure S21: SEM images of differentiated cells cultured on the PDMS-transferred ME mesh: (a) with AMF application and (b) without AMF application.

βIII-tubulin						
		w/o AMF			w/AMF	
G1	0.267	0.358	0.403	0.237	0.363	0.495
G2	0.144	0.174	0.292	1.604	4.967	2.079
G3	0.359	0.158	0.376	6.845	4.124	4.463
G4	0.282	0.135	0.396	6.033	6.397	4.625
G5	0.187	0.265	0.284	12.997	10.835	11.475

MAP2						
		w/o AMF			w/AMF	
G1	0.256	0.368	0.429	0.268	0.263	0.435
G2	0.357	0.297	0.254	1.807	1.014	2.935
G3	0.146	0.211	0.179	3.467	3.831	2.026
G4	0.189	0.167	0.155	2.015	4.402	4.136
G5	0.316	0.166	0.17	13.269	14.46	13.554

Figure S22: Quantitative assessment data through mean fluorescence intensity across all samples.

References

[1] M. Kim, D. Kim, B. Aktas, H. Choi, J. Puigmartí-Luis, B. J. Nelson, S. Pané, X.-Z. Chen, *Adv. Mater. Technol.* **2023**, *8*, 2202097.

[2] M. Kim, D. Kim, M. Mirjolet, N. A. Shepelin, T. Lippert, H. Choi, J. Puigmartí-Luis, B. J. Nelson, X.-Z. Chen, S. Pané, *Adv. Mater.* **2024**, *36*, 2404825.
